# Remyelination of chronic demyelinated lesions with directly induced neural stem cells

**DOI:** 10.1093/brain/awaf208

**Published:** 2025-07-07

**Authors:** Luca Peruzzotti-Jametti, Nunzio Vicario, Giulio Volpe, Sandra Rizzi, Cheek Kwok, Ivan Lombardi, Mads S Bergholt, Lucas Barea-Moya, Andrea D’Angelo, Alexandra M Nicaise, Giuseppe D’Amico, Grzegorz Krzak, Cory M Willis, Sara Gil-Perotin, Olena Hruba, Alice Braga, Molly M Stevens, José M Garcia-Verdugo, Kourosh Saeb-Parsy, Chao Zhao, Robin J M Franklin, Frank Edenhofer, Stefano Pluchino

**Affiliations:** Department of Clinical Neurosciences and NIHR Biomedical Research Centre, University of Cambridge, Cambridge CB2 0AH, UK; Department of Clinical Neurosciences and NIHR Biomedical Research Centre, University of Cambridge, Cambridge CB2 0AH, UK; Department of Biomedical and Biotechnological Sciences, Physiology Section, University of Catania, Catania I-95123, Italy; Department of Clinical Neurosciences and NIHR Biomedical Research Centre, University of Cambridge, Cambridge CB2 0AH, UK; Institute of Molecular Biology & CMBI, Genomics, Stem Cell Biology & Regenerative Medicine, Leopold-Franzens-University Innsbruck, Innsbruck 6020, Austria; Institute of Anatomy and Cell Biology, University of Würzburg, Würzburg 97070, Germany; Department of Clinical Neurosciences and NIHR Biomedical Research Centre, University of Cambridge, Cambridge CB2 0AH, UK; Department of Biotechnology and Biosciences, University of Milano-Bicocca, Milano 20126, Italy; Centre for Craniofacial & Regenerative Biology, King’s College London, London WC2R 2LS, UK; Department of Materials, Department of Bioengineering, Institute of Biomedical Engineering, Imperial College London, London SW7 2AZ, UK; Laboratory of Comparative Neurobiology, Cavanilles Institute of Biodiversity and Evolutionary Biology, University of Valencia and CIBERNED-ISCIII, Valencia 46980, Spain; Research Group in Immunotherapy and Biomodels of Autoimmunity, Health Research Institute La Fe, Valencia 46026, Spain; Department of Neurology, Hospital Universitario y Politécnico La Fe, Valencia 46026, Spain; Department of Clinical Neurosciences and NIHR Biomedical Research Centre, University of Cambridge, Cambridge CB2 0AH, UK; Department of Clinical Neurosciences and NIHR Biomedical Research Centre, University of Cambridge, Cambridge CB2 0AH, UK; Department of Clinical Neurosciences and NIHR Biomedical Research Centre, University of Cambridge, Cambridge CB2 0AH, UK; Department of Clinical Neurosciences and NIHR Biomedical Research Centre, University of Cambridge, Cambridge CB2 0AH, UK; Department of Clinical Neurosciences and NIHR Biomedical Research Centre, University of Cambridge, Cambridge CB2 0AH, UK; Laboratory of Comparative Neurobiology, Cavanilles Institute of Biodiversity and Evolutionary Biology, University of Valencia and CIBERNED-ISCIII, Valencia 46980, Spain; Research Group in Immunotherapy and Biomodels of Autoimmunity, Health Research Institute La Fe, Valencia 46026, Spain; Red Española de Terapias Avanzadas, TERAV-RICORS, RD24/0014/0009, Instituto de Salud Carlos III, Sevilla 41092, Spain; Department of Clinical Neurosciences and NIHR Biomedical Research Centre, University of Cambridge, Cambridge CB2 0AH, UK; Department of Clinical Neurosciences and NIHR Biomedical Research Centre, University of Cambridge, Cambridge CB2 0AH, UK; Department of Materials, Department of Bioengineering, Institute of Biomedical Engineering, Imperial College London, London SW7 2AZ, UK; Laboratory of Comparative Neurobiology, Cavanilles Institute of Biodiversity and Evolutionary Biology, University of Valencia and CIBERNED-ISCIII, Valencia 46980, Spain; Department of Surgery, Cambridge Biomedical Research Centre, University of Cambridge, Cambridge CB2 0QQ, UK; Wellcome Trust-Medical Research Council Cambridge Stem Cell Institute, Jeffrey Cheah Biomedical Centre, University of Cambridge, Cambridge CB2 0AW, UK; Wellcome Trust-Medical Research Council Cambridge Stem Cell Institute, Jeffrey Cheah Biomedical Centre, University of Cambridge, Cambridge CB2 0AW, UK; Institute of Molecular Biology & CMBI, Genomics, Stem Cell Biology & Regenerative Medicine, Leopold-Franzens-University Innsbruck, Innsbruck 6020, Austria; Institute of Anatomy and Cell Biology, University of Würzburg, Würzburg 97070, Germany; Department of Clinical Neurosciences and NIHR Biomedical Research Centre, University of Cambridge, Cambridge CB2 0AH, UK

**Keywords:** iNSC grafts, remyelination, multiple sclerosis, oligodendrocyte progenitor cells, demyelination, transplantation

## Abstract

The limited ability of CNS progenitor cells to differentiate into oligodendrocytes limits the repair of demyelinating lesions and contributes to the disability of people with progressive multiple sclerosis (PMS). Neural stem cell (NSC) transplantation has emerged as a safe therapeutic approach in people with PMS, where it holds the promise of healing the injured CNS. However, the mechanisms by which NSC grafts could promote CNS remyelination need to be carefully assessed before their widespread clinical adoption.

In this study, we used directly induced NSCs (iNSCs) as a novel transplantation source to boost remyelination in the CNS. Using a mouse model of focal lysophosphatidylcholine (LPC)-induced demyelination, we found that mouse iNSCs promote remyelination by enhancing endogenous oligodendrocyte progenitor cells differentiation and by directly differentiating into mature oligodendrocytes. Transplantation of mouse iNSCs in LPC-lesioned *Olig1^−/−^* mice, which exhibits impaired remyelination, confirmed the direct remyelinating ability of grafts and the formation of new exogenous myelin sheaths. We also demonstrated that the xenotransplantation of human iNSCs (hiNSCs) is safe in mice, with hiNSCs persisting long-term in demyelinating lesions where they can produce graft-derived human myelin.

Our findings support the use of NSC therapies to enhance remyelination in chronic demyelinating disorders, such as PMS.

## Teaser

Peruzzotti-Jametti *et al.* examine the remyelination potential of mouse- and human-derived directly induced neural stem cell (iNSC) grafts. They show that iNSCs can differentiate into mature oligodendrocytes and remyelinate axons in demyelinated lesions, highlighting their potential to promote remyelination in multiple sclerosis.

## Introduction

Multiple sclerosis (MS) is an immune mediated demyelinating disorder of the CNS and the most common cause of neurological disability in young adults.^1^ Demyelination in MS is the result of complex interactions between the immune system and the CNS, which evolve over the disease course and a patient's lifetime.^2^ In the early phase of MS, when infiltrating lymphocytes cause most of the CNS damage, the endogenous pool of oligodendrocyte progenitor cells (OPCs) can partially remyelinate demyelinating lesions by differentiating into mature, myelin-forming, oligodendrocytes (OLs). However, in the later chronic progressive stage of disease, this endogenous remyelination ability is severely insufficient.^3^ This leads to inadequate tissue repair and increasing lesion load, leading to the accumulation of disability seen in people with progressive multiple sclerosis (PMS).

Recent phase I clinical trials have demonstrated the safety and feasibility of neural stem cell (NSC) transplantation in people with PMS,^4,5^ paving the way for further research investigating its full therapeutic potential. Previous data obtained in preclinical models of MS-like diseases have shown that transplanted NSCs can significantly ameliorate clinico-pathological deficits of disease by exerting beneficial immunomodulatory and neurotrophic effects on the CNS.^6,7^ However, NSC grafts have shown a limited ability to replace damaged OLs and to generate new myelin *in vitro*, or when transplanted into highly inflammatory micro-environments.^8^ Stably expandable directly induced NSCs (iNSCs) from patients’ skin fibroblasts offer an alternative source of NSCs for transplantation in people with PMS.^8-10^ This approach provides a rapid and scalable method for generating NSCs that circumvents ethical concerns and the need for immunosuppression.^7^ However, the effectiveness of these grafts to promote endogenous remyelination and/or directly producing new exogenous myelin remains unclear.

Our study aims to explore the potential of NSC grafts in promoting the remyelination of demyelinating lesions upon local intraparenchymal transplantation in the spinal cord. Through a comprehensive assessment of the differentiation ability of mouse NCS, mouse iNSCs and human iNSCs, we provide new evidence supporting the use of CNS specific transplantation therapies in chronic demyelinating disorders, such as PMS.

## Materials and methods

### Cell lines and *in vitro* culture

Details of all cell lines, their origin, and *n* per treatment group are reported in Supplementary Table 1. Prior to all transplantation studies, cell viability was evaluated with trypan blue exclusion (>91% in all cell lines) and mycoplasma negative cells at passage *n* ≤ 30 were used in all experiments. Further details are available in the Supplementary material, ‘Methods’ section (including the lentiviral fGFP tagging).

### Animals, focal demyelination of the spinal cord and cell transplantation

Spinal cord demyelinating lesions were induced in 8/12-week-old wild-type (WT) C57BL/6 or *Olig1*^−/−^ mice by stereotaxic injection of 1 μl of 1% lysophosphatidylcholine (LPC, L4129 Sigma-Aldrich) between the T12 and T13 vertebrae (using a Hamilton syringe) reaching the ventral spinal cord, as previously described.^11^ At 3 days post lesion (dpl), mice were randomized to receive either 1 × 10^5^ cells in 1 μl of PBS or an equal volume of the vehicle solution for PBS-treated mice. Further details of the methods [including *ex vivo* spinal cord histopathology, PCR, immunoblotting, *in situ* hybridization, transmission electron microscopy (TEM) and pre-embedding immunogold labelling, confocal Raman microspectroscopy, teratoma assay and statistical analyses] are available in the Supplementary material, ‘Methods’ section.

## Results

### iNSC grafts integrate into the demyelinated spinal cord

**Figure 1:**
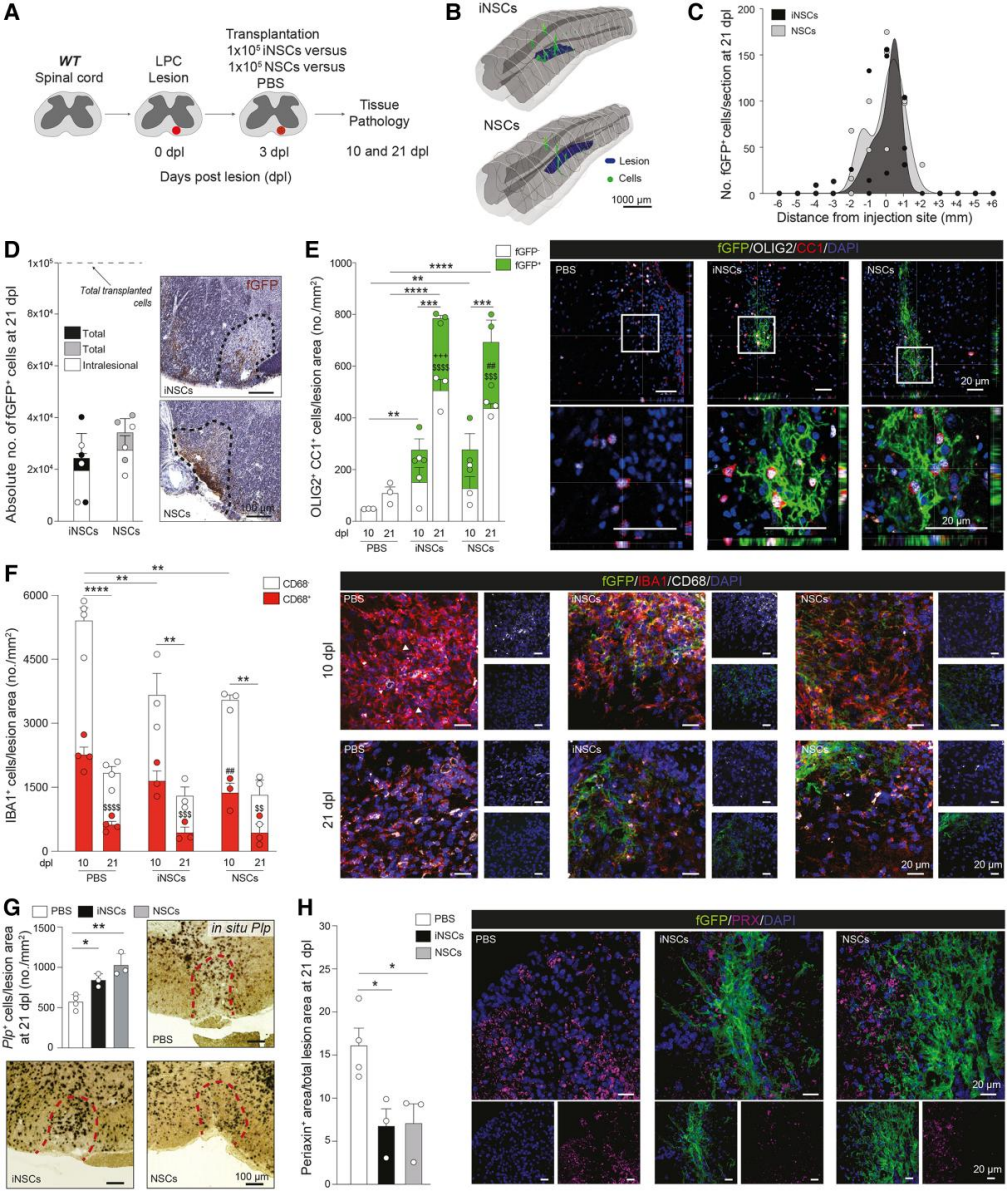
**Transplanted mouse NSCs and induced NSCs survive, integrate and differentiate into fully mature oligodendrocytes in LPC spinal cord lesions of wild-type C57BL/6 mice.** (**A**) Experimental setup for LPC-induced lesions and transplantation of mouse NSCs and iNSCs in wild-type (WT) C57BL/6 mice. (**B**) Representative 3D reconstructions showing individual transplanted fGFP^+^ cells (green shaded areas) in relation to the LPC-induced lesions of the ventral white matter of the spinal cord (blue shaded areas). (**C** and **D**) Stereology-based quantification of cell survival showing the distribution of fGFP^+^ iNSCs and fGFP^+^ NSCs (**C**) and absolute numbers of cells (total number and intralesional) with representative images (**D**). Data are mean values ± SEM analysed in *n* = 3 mice/group. (**E**) Quantification and representative images of endogenous (fGFP^−^) and exogenous (fGFP^+^) OLIG2^+^/CC1^+^ mature oligodendrocytes found in LPC-induced lesions of PBS-, iNSC- and NSC-treated mice. Data are mean values ± SEM analysed in *n* = 3 mice/group/time point. **P ≤ 0.01, ***P ≤ 0.001, ****P ≤ 0.0001; ^$$$^*P* ≤ 0.001, ^$$$$^*P* ≤ 0.0001 versus PBS at 21 dpl; ^+++^*P* ≤ 0.001 versus GFP^−^ iNSC at 10 dpl; ^##^*P* ≤ 0.01 versus GFP^−^ NSC at 10 dpl. Two-way ANOVA, Tukey's multiple comparisons test. (**F**) Quantification and representative images of IBA1^+^ myeloid response and relative CD68 expression found in LPC-induced lesions of PBS-, iNSC- and NSC-treated mice. Data are mean values ± SEM analysed in *n* = 4, *n* = 3 and *n* = 3 mice, respectively. **P ≤ 0.01, ****P ≤ 0.0001; ^$$^*P* ≤ 0.05, ^$$$^*P* ≤ 0.001, ^$$$$^*P* ≤ 0.0001 comparing IBA1^+^/CD68^+^ between 21 dpl versus 10 dpl in the same group; ^##^*P* ≤ 0.01 versus PBS IBA1^+^/CD68^+^ at 21 dpl. Two-way ANOVA, Tukey's multiple comparisons test. (**G**) Quantification and representative images of *in situ* hybridization for *Plp* from PBS-, iNSC- and NSC-treated mice. Data are mean values ± SEM analysed in *n* = 4, *n* = 3 and *n* = 3 mice, respectively. *P ≤ 0.05, **P ≤ 0.01. One-way ANOVA, Holm-Šídák's multiple comparison test. (**H**) Quantification and representative images of PRX^+^ area of PBS-, iNSC- and NSC-treated mice showing stem cell contribution to remyelinating the LPC-induced spinal cord lesions. Data are mean area/total lesion area ± SEM analysed in *n* = 4, *n* = 3, *n* = 3 mice, respectively. *P ≤ 0.05. One-way ANOVA, Holm-Šídák's multiple comparison test. dpl = days post lesion; iNSC = induced neural stem cell; LPC = lysophosphatidylcholine; NSC = neural stem cell; SEM = standard error of the mean.

To model CNS demyelination, we induced a focal lesion in the ventrolateral white matter of the spinal cord of C57BL/6 wild-type mice via a local LPC injection.^11^ At 3 dpl, mice received an intralesional injection of either mouse syngeneic fGFP^+^ iNSCs (iNSCs-treated), fGFP^+^ NSCs (NSCs-treated) or the vehicle solution (PBS-treated). Tissues were then collected at 10 and 21 dpl for downstream analyses (Fig. 1A).

At 21 dpl, both iNSCs and NSCs successfully integrated into the demyelinated spinal cord, with cell density progressively decreasing from the injection site (Fig. 1B and C). A total of 24.3% ± 9.5% and 34.2% ± 5.4% transplanted cells survived at 21 dpl in iNSCs-treated and in NSCs-treated mice, respectively, with similar percentages of iNSCs and NSCs found intralesionally (Fig. 1D). At 21 dpl, most transplanted cells expressed SOX1 (71.4% ± 4.8% and 66.5% ± 4.5%), while only a minority (1.3% ± 0.7% and 0.5% ± 0.3%) expressed the proliferation marker KI67 (Supplementary Fig. 1A and B). Both cellular grafts expressed the glial marker GFAP (18.6% ± 2.5% and 23.3% ± 4.1%) and, to a lower extent, the neuronal marker TUJ1 (8.9% ± 3.2% and 7.7% ± 2.7%).

When investigating the expression of markers indicative of oligodendroglial differentiation, we found that at 21 dpl, the subset of iNSCs and NSCs expressing the OPC markers NG2^+^/OLIG2^+^ (2.9% ± 0.3% and 4.0% ± 0.6%) was significantly lower than those expressing the mature OL markers OLIG2^+^/CC1^+^ (8.5% ± 1.3% and 11.5% ± 1.8%), which was 2.0-fold higher compared to an earlier time point (10 dpl) (Supplementary Fig. 1C and D), suggesting an increased differentiation of the grafts into OLs over time.

### iNSC grafts increase the number of endogenous OLs and promote faster resolution of inflammatory myeloid responses

We next assessed how stem cell transplantation affected the remyelinating response after LPC injury by analysing the number of endogenous (fGFP^−^) versus exogenous (fGFP^+^) OLIG2^+^/CC1^+^ mature OLs at 10 and 21 dpl, intralesionally.

Both iNSCs-treated and NSCs-treated wild-type mice displayed an increase in endogenous fGFP^−^ mature OLs versus control PBS-treated mice at 21 dpl (Fig. 1E). This enhanced endogenous response was coupled with a direct intralesional differentiation of the grafts into fGFP^+^/OLIG2^+^/CC1^+^ cells, which significantly increased the total number of OLs intralesionally versus PBS-treated mice (5.8-fold at 10 dpl and 7.2- and 6.4-fold at 21 dpl).

Since early myeloid cell activation in the LPC lesion model is known to exert anti-regenerative effects and limit remyelination,^12^ we next examined the effects of grafts on microglia/macrophage numbers and polarisation (Fig. 1F). At 10 dpl, both iNSCs-treated and NSCs-treated wild-type mice showed a significant 1.5-fold reduction of total IBA1^+^ cells and IBA1^+^/CD68^+^ cells (1.4-fold and 1.6-fold) versus PBS-treated mice. At 21 dpl, the number of total IBA1^+^ and IBA1^+^/CD68^+^ cells was significantly reduced versus 10 dpl in all treatment groups.

*In situ* hybridization showed that both grafts induced a significant increase in the expression of *Plp*^+^ cells at 21 dpl intralesionally versus PBS-treated mice (Fig. 1G), consistently with the increased number of total OLIG2^+^/CC1^+^ OLs.

Since previous data have shown that remyelination after demyelination of the spinal cord depends on both OLs derived from CNS-resident OPCs and Schwann cells (SCs) that originate from OPCs or the peripheral nervous system (PNS),^11^ we next assessed the involvement of stem cells after transplantation. Both iNSCs-treated and NSCs-treated mice showed a significant reduction of PRX^+^, a myelin protein specific to the PNS that is absent in CNS myelin,^13^ versus PBS-treated mice at 21 dpl (Fig. 1H).

Altogether these findings suggest that both iNSC and NSC grafts foster remyelination in the injured spinal cord by exerting early immunomodulatory effects and reducing the contribution of stem cells, thus promoting more advantageous CNS specific remyelination^14^ through enhanced endogenous OPC differentiation and direct differentiation into mature OLs.

### iNSC grafts promote OL maturation and resolution of myeloid responses in *Olig1*^−/−^ demyelinated spinal cord

**Figure 2:**
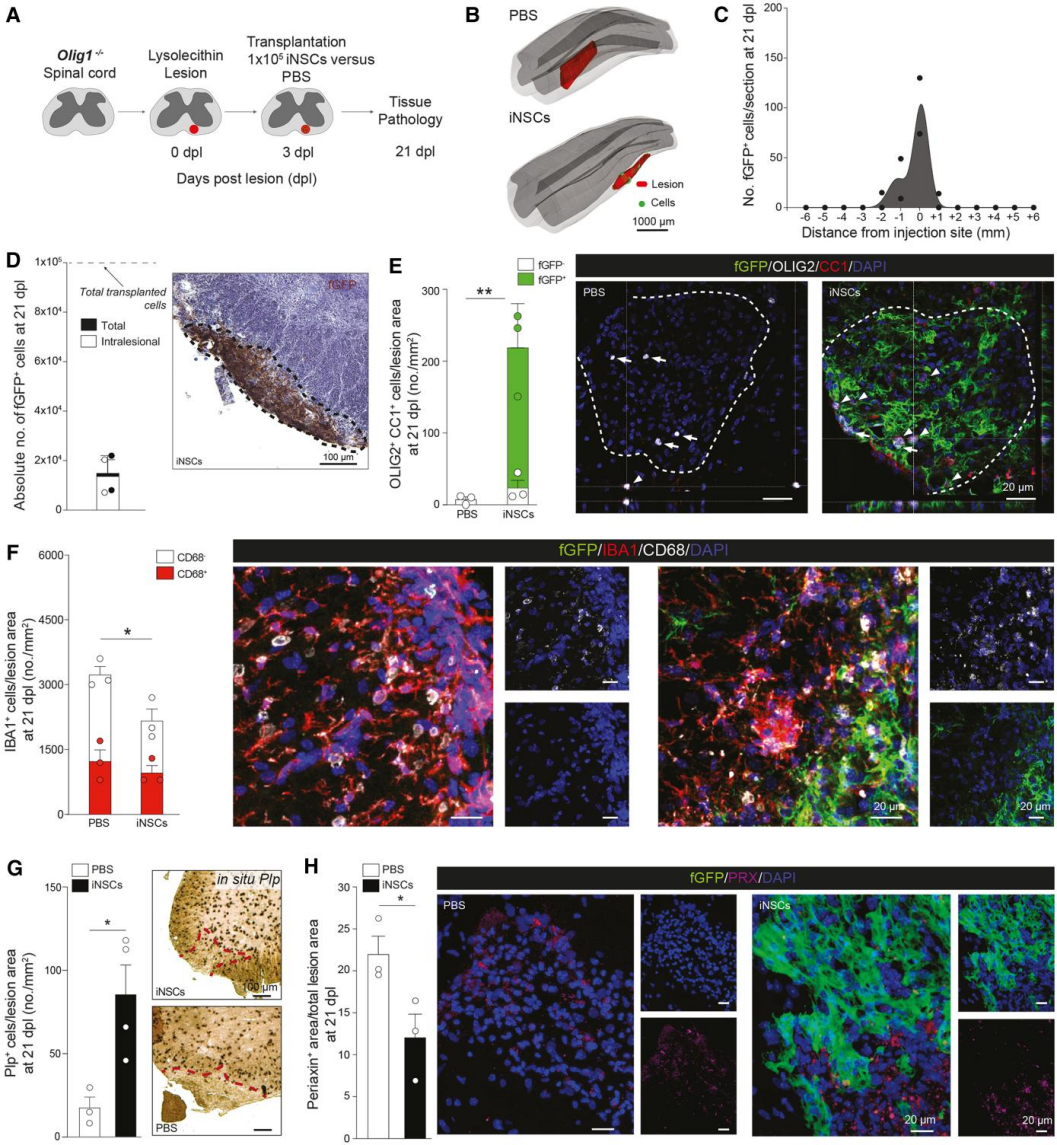
**Transplanted mouse induced neural stem cells survive, integrate and generate mature oligodendrocytes in LPC spinal cord lesions of *Olig1*^−/−^ mice.** (**A**) Experimental setup for LPC-induced lesions and transplantation of mouse iNSCs in *Olig1*^−/−^ mice. (**B**) Representative 3D reconstructions showing individual transplanted fGFP^+^ iNSCs (green shaded areas) in relation to the LPC-induced lesions of the ventral white matter of the spinal cord (red shaded areas). (**C** and **D**) Stereology-based quantification of cell survival showing the distribution of fGFP^+^ iNSCs (**C**) and absolute numbers of cells (total number and intralesional) with representative images (**D**). Data are mean values ± SEM analysed in *n* = 2 mice. (**E**) Quantification and representative images of endogenous (fGFP^−^) and exogenous (fGFP^+^) OLIG2^+^/CC1^+^ mature oligodendrocytes found in LPC-induced lesions of PBS- and iNSC-treated *Olig1*^−/−^ mice at 21 dpl. Data are mean values ± SEM analysed in *n* = 3 mice/group. **P ≤ 0.01. Unpaired *t*-test. (**F**) Quantification and representative images of IBA1^+^ myeloid response and relative CD68 expression found in LPC-induced lesions of PBS- and iNSC-treated *Olig1*^−/−^ mice at 21 dpl. Data are mean values ± SEM analysed in *n* = 3 mice/group. *P ≤ 0.05. Unpaired *t*-test. (**G**) Quantification and representative images of *in situ* hybridization for *Plp* from PBS- and iNSC-treated *Olig1*^−/−^ mice at 21 dpl. Data are mean values ± SEM analysed in *n* = 3, *n* = 4 mice, respectively. *P ≤ 0.05. Unpaired *t*-test. (**H**) Quantification and representative images of PRX^+^ area of PBS- and iNSC-treated *Olig1*^−/−^ mice showing stem cell contribution to remyelinating LPC-induced spinal cord lesions at 21 dpl. Data are mean area/total lesion area ± SEM analysed in *n* =3 mice/group. *P ≤ 0.05. Unpaired *t*-test. dpl = days post lesion; iNSC = induced neural stem cell; LPC = lysophosphatidylcholine;SEM = standard error of the mean.

After observing comparable features of iNSC and NSC grafts *in vivo*, we next sought to investigate the direct role of iNSCs in CNS remyelination by transplanting them in a mouse model that displays impaired endogenous OL differentiation after injury. To this aim, we used *Olig1*^−/−^ mice (Supplementary Fig. 2A and B), which have normal endogenous OPC recruitment, but severely impaired OL differentiation in response to CNS injury.^15,16^ To assess the impact of the grafts on the incomplete remyelination of these transgenic mice, we focused on 21 dpl, a time point when WT mice exhibit instead a complete remyelination (Fig. 2A).

We found that iNSCs successfully integrated in the lesioned spinal cord of *Olig1*^−/−^ mice, with cell density progressively decreasing from the injection site (Fig. 2B and C). A total of 15.0% ± 5.7% of transplanted iNSCs survived in the lesioned spinal cord at 21 dpl, with 13.7% ± 5.4% of cells found intralesionally (Fig. 2D).

Quantification of cell markers revealed that most transplanted cells expressed SOX1 (68.7% ± 2.0%), while only a minority of iNSCs were proliferating (2.0% ± 0.8%) (Supplementary Fig. 2C). Further *in vivo* differentiation profiling showed that iNSCs were expressing GFAP (12.6% ± 0.8%), TUJ1 (3.2% ± 1.1%), NG2/OLIG2 (1.2% ± 0.8%) and OLIG2/CC1 (9.0% ± 1.9%), similar to our results from wild-type transplanted mice.

We further investigated how iNSC transplantation contributed to the remyelinating response by assessing the number of endogenous and exogenous OLIG2^+^/CC1^+^ mature OLs at 21 dpl (Fig. 2E). We found that endogenous fGFP^−^/OLIG2^+^/CC1^+^ OLs in both iNSC-treated mice and PBS-treated at 21 dpl were very low (23.3 ± 10.5 cells/mm^2^ versus 7.2 ± 3.6 cells/mm^2^), as expected.^15,16^ On the contrary, we found that a total of 196.07 ± 34.78 iNSCs/mm^2^ differentiated into fGFP^+^/OLIG2^+^/CC1^+^ OLs, leading to a significant 30.4-fold increase in the total number of mature OLs versus PBS-treated *Olig1*^−/−^ mice (219.4 ± 39.3 versus 7.2 ± 3.6 cells/mm^2^).

When assessing myeloid cell reaction, we found that iNSC grafts induced a 1.4-fold significant reduction of total IBA1^+^ (1909 ± 197.2 cells/mm^2^) versus PBS-treated *Olig1*^−/−^ mice (2679 ± 229.1 cells/mm^2^) (Fig. 2F). *In situ* hybridization showed that the number of *Plp*^+^ cells was significantly increased in iNSC-treated versus PBS-treated *Olig1*^−/−^ mice (85.4 ± 17.6 cells/mm^2^ versus 17.4 ± 6.3 cells/mm^2^) (Fig. 2G), while we found a significant reduction of PRX in iNSC-treated versus PBS-treated *Olig1*^−/−^ mice (12.06% ± 2.77% versus 21.98% ± 2.14%) (Fig. 2H).

Hence, iNSC grafts show immunomodulatory effects in the chronically demyelinated spinal cord white matter of *Olig1*^−/−^ mice, where they reduce the contribution of stem cells, and directly differentiate into myelin forming cells.

### iNSC grafts directly remyelinate the chronically demyelinated spinal cord of *Olig1*^−/−^ mice

To assess the extent of remyelination induced by iNSC grafts, we next conducted ultrastructural TEM analyses of the LPC-lesioned spinal cords of iNSC-treated *Olig1*^−/−^ mice, PBS-treated *Olig1*^−/−^ mice and PBS-treated wild-type controls at 21 dpl.

**Figure 3:**
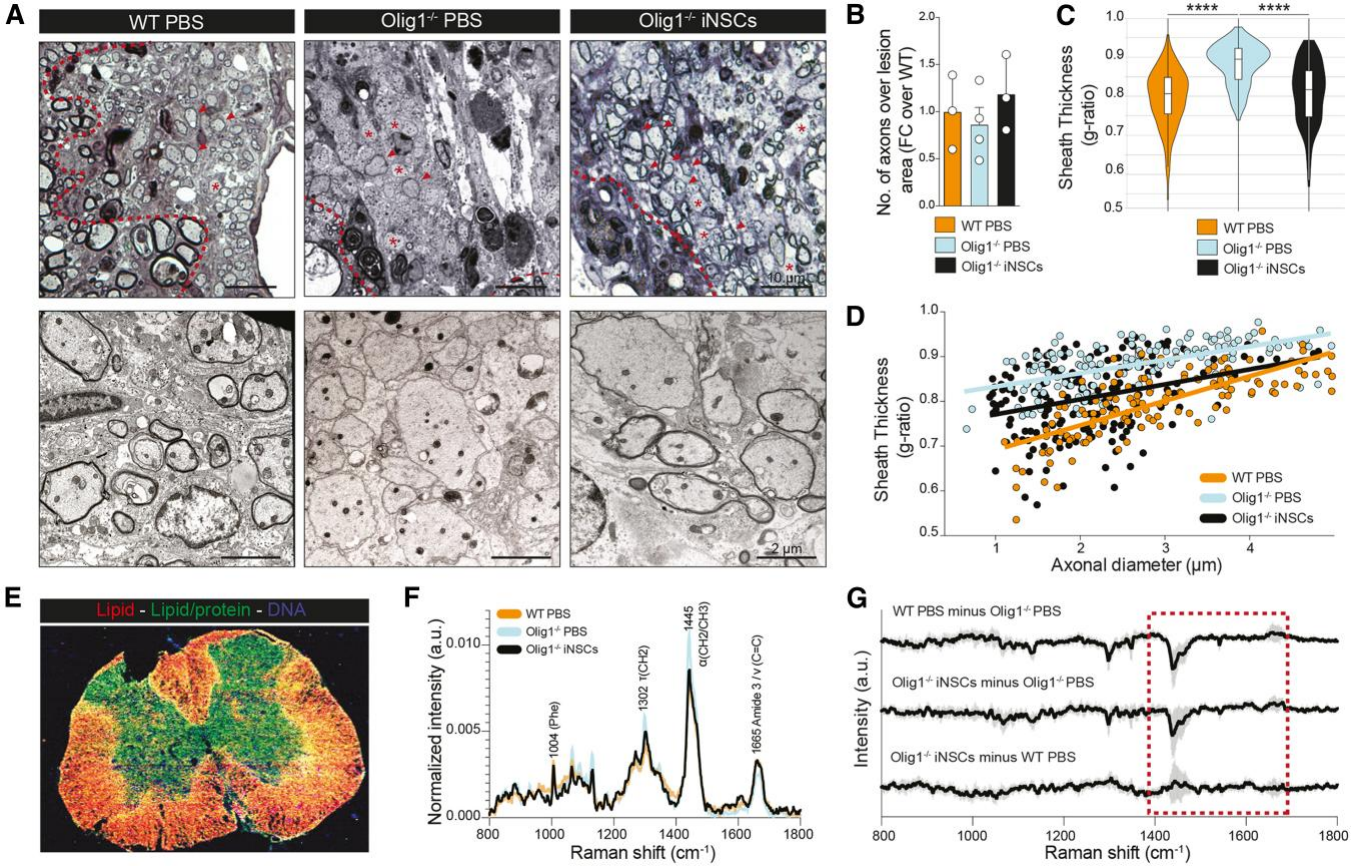
**Transplanted mouse induced neural stem cells remyelinate LPC lesions of *Olig1*^−/−^ mice.** (**A**) Representative pictures of toluidine blue stained LPC lesions (*top*) and corresponding TEM images (*bottom*) of PBS-treated wild-type (WT) mice, PBS-treated *Olig1*^−/−^ mice and iNSC-treated *Olig1*^−/−^ mice. Asterisks indicate demyelinated axons, arrowheads indicate remyelinated axons, dashed lines indicate the border of the lesion. (**B**) Quantification of the total number of axons per lesion area in PBS-treated WT mice (*n* = 3), PBS-treated *Olig1*^−/−^ mice (*n* = 4) and iNSC-treated *Olig1*^−/−^ mice (*n* = 3). (**C** and **D**) Quantification of the g-ratio of axons within the demyelinated lesion of PBS-treated WT mice, PBS-treated *Olig1*^−/−^ mice and iNSC-treated *Olig1*^−/−^ mice. Data are mean ± min max values (**C**) and single values plotted on axonal diameter (**D**). ****P ≤ 0.0001. One way ANOVA, Holm-Šídák's multiple comparisons test. (**E**) Confocal Raman imaging of a representative LPC lesion (from PBS-treated *Olig1*^−/−^ mouse) at 21 dpl, showing peaks associated with lipids (1445 cm^−1^), lipid/protein (1665 cm^−1^) and DNA (1345 cm^−1^). (**F**) Mean Raman spectra of lesions in PBS-treated WT (*n* = 3), PBS-treated *Olig1*^−/−^ (*n* = 5) and iNSC-treated *Olig1*^−/−^ mice (*n* = 3) at 21 dpl. Peaks associated with myelin/lipids [1302 cm⁻^1^ τ(CH2), 1445 cm⁻^1^ α(CH2/CH3) and 1665 cm⁻^1^ ν(C = C)] or axons/proteins [e.g. 1004 (phenylalanine)] are labelled. (**G**) Mean difference spectra (±1 standard deviation) of LPC lesions at 21 dpl, comparing the three different groups: PBS-treated WT (*n* = 3), PBS-treated *Olig1*^−/−^ (*n* = 5) and iNSC-treated *Olig1*^−/−^ (*n* = 3) mice. PBS-treated WT and iNSC-treated *Olig1*^−/−^ minus PBS-treated *Olig1*^−/−^ difference spectra (*top two lines*) show major differences, while PBS-treated WT and iNSC-treated *Olig1*^−/−^ difference spectra (*bottom line*) are superimposable. Red dotted box outlines peaks associated with myelin/lipids. dpl = days post lesion; iNSC = induced neural stem cell; LPC = lysophosphatidylcholine; min = minimum; max = maximum; TEM = transmission electron microscopy.

PBS-treated *Olig1*^−/−^ mice exhibited a sustained extensive demyelination of LPC lesions, in line with a severe delay of their remyelination processes^15,16^ (Fig. 3A). Instead, iNSC-treated *Olig1*^−/−^ mice showed active remyelination of lesions, which was superimposable to the spontaneous remyelinating response seen in wild-type mice. While we did not detect striking features of axonal degeneration, nor significant changes in the number of axons among treatment groups (Fig. 3B), we found a significant decrease of axonal g-ratios in iNSC-treated *Olig1*^−/−^ mice (0.80 ± 0.006) versus PBS-treated *Olig1*^−/−^ mice (0.88 ± 0.004), almost reaching values observed in PBS-treated wild-type mice (0.79 ± 0.006; Fig. 3C and D).

We further analysed the molecular composition of LPC lesions at 21 dpl, using confocal Raman microspectroscopy (Fig. 3E), an approach that has high sensitivity to myelin [particularly around 1302 cm⁻^1^ τ(CH2), 1445 cm⁻^1^ α(CH2/CH3) and 1665 cm⁻^1^ ν(C = C) of lipids].^17^ The mean Raman spectra showed no significant differences in protein related peaks [e.g. 1004 (phenylalanine) or 1227–1272 (Amide III)] associated with axons^17^ (Fig. 3F). We then analysed the mean difference spectra (±1 standard deviation) and found instead that both PBS-treated wild-type and iNSC-treated *Olig1*^−/−^ mice displayed a very different molecular lesional profile versus PBS-treated Olig1^−/−^ mice, especially in Raman peaks associated with myelin (Fig. 3G). On the contrary, the lesional myelin composition of iNSC-treated *Olig1*^−/−^ mice closely resembled the spontaneous remyelination of PBS-treated wild-type mice (Fig. 3G), with higher myelin index versus PBS-treated Olig1^−/−^ mice (Supplementary Fig. 3).

Altogether, these data show that iNSCs have the potential to directly differentiate in OLs and drive myelin formation, even when transplanted in lesions where endogenous remyelinating processes are chronically impaired.

### Human iNSC xenografts are safe and survive in chronically demyelinated spinal cord of *Olig1*^−/−^ mice

We next reprogrammed human iNSCs (hiNSCs) from dermal fibroblasts^10^ for xenotransplantation studies (Supplementary Fig. 4A). Prior to transplantation, fGFP^+^ hiNSCs (Supplementary Fig. 4B) were negative for the pluripotency marker Oct4 (Supplementary Fig. 4C), while showing sustained expression of both SOX1 and SOX2 *in vitro* (Supplementary Fig. 4D).

**Figure 4:**
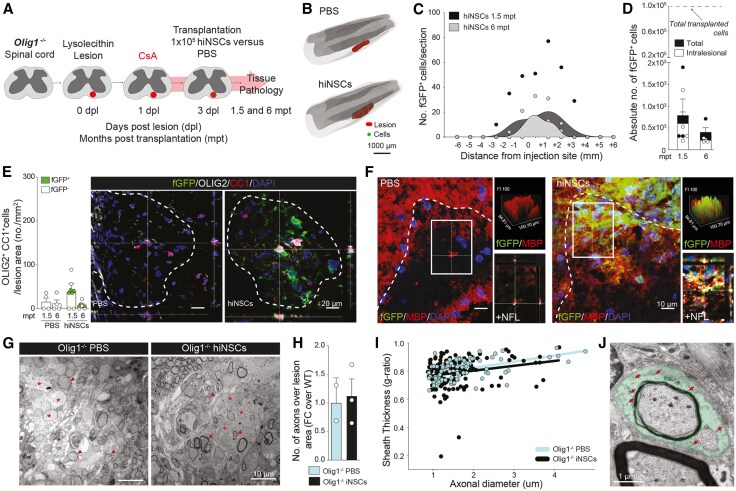
**Transplanted human induced neural stem cells survive, integrate and differentiate in mature oligodendrocytes in LPC spinal cord lesions of *Olig1*^−/−^ mice.** (**A**) Experimental setup for LPC-induced lesions and transplantation of hiNSCs in *Olig1*^−/−^ mice. (**B**) Representative 3D reconstructions showing individual transplanted fGFP^+^ hiNSCs (green shaded areas) in relation to the LPC-induced lesions of the ventral white matter of the spinal cord (red shaded areas). (**C** and **D**) Stereology-based quantification of cell survival showing distribution of fGFP^+^ hiNSCs (**C**) with absolute numbers of cells (total number and intralesional) (**D**). Data are mean values ± SEM analysed in *n* = 4 mice at 1.5 mpt and *n* = 4 mice at 6 mpt. (**E**) Quantification and representative images of endogenous (fGFP^−^) and exogenous (fGFP^+^) OLIG2^+^/CC1^+^ mature oligodendrocytes found in LPC-induced lesions of PBS- and hiNSC-treated mice. Data are mean values ± SEM analysed in *n* = 4 mouse/group/time point. (**F**) Representative images of immunofluorescence stainings for MBP (myelin marker) and fGFP^+^ within lesions (maximum projections are shown on the *left*). *Right*: Respective qualitative histograms of the lesions (*top*) and single *z*-stack of the inset shown in the maximum projection with added NFL staining for axons (*bottom*). (**G**) Representative TEM images of LPC lesions from PBS-treated *Olig1*^−/−^ and hiNSC-treated *Olig1*^−/−^ mice at 6 mpt. Asterisks indicate demyelinated axons, arrowheads indicate remyelinated axons. (**H**) Quantification of the total number of axons per lesion area in PBS-treated *Olig1*^−/−^ mice (*n* = 2) and hiNSC-treated *Olig1*^−/−^ mice (*n* = 3). (**I**) Quantification of the g-ratio of axons within the demyelinated lesion of PBS-treated *Olig1*^−/−^ mice (*n* = 2) and hiNSC-treated *Olig1*^−/−^ mice (*n* = 3). Data are single values plotted on axonal diameter. (**J**) Representative TEM image showing fGFP^+^ hiNSCs (pseudocoloured in green) wrapping around an axon within LPC lesions. Arrows show immunogold labelling for fGFP. hiNSC = human induced neural stem cell; LPC = lysophosphatidylcholine; mpt = months post transplantation; SEM = standard error of the mean; TEM = transmission electron microscopy; WT = wild-type.

We first transplanted fGFP^+^ hiNSCs, or control fGFP^+^ hiPSCs, under the kidney capsule of NOD SCID mice,^18^ to assess their tumorigenicity *in vivo*. While hiPSC-treated mice showed consistent teratoma formation at 70 dpt, no teratomas were ever seen in the hiNSC-treated group (Supplementary Fig. 5A–C). We next investigated hiNSC grafts survival and integration in the lesioned spinal cord of immunosuppressed *Olig1*^−/−^ mice (Fig. 4A).

We found that hiNSC grafts were able to persist in the spinal cord of all transplanted mice at 1.5- and 6-months post transplantation (mpt), with cell density progressively decreasing from the injection site (Fig. 4B and C). However, compared to mouse iNSCs, the survival rate of hiNSCs in the mouse spinal cord was relatively low (0.8% ± 0.4% and 0.4% ± 0.1% of transplanted cells at 1.5 and 6 mpt, respectively) (Fig. 4D).

Nonetheless, we found that hiNSC xenografts were able differentiate into mature OLs intralesionally (2.8 ± 1.6 fGFP^+^/OLIG2^+^/CC1^+^ cells/mm^2^ found in *n* = 2/4 mice at 1.5 mpt; and 1.1 fGFP^+^/OLIG2^+^/CC1^+^ cells/mm^2^ found in *n* = 1/4 mice at 6 mpt) (Fig. 4E), where human fGFP^+^ MBP^+^ myelin was also observed (Fig. 4F).

Ultrastructurally, TEM analyses of hiNSC-treated *Olig1*^−/−^ mice and PBS-treated *Olig1*^−/−^ mice showed incomplete remyelination of LPC spinal cord lesions at 6 mpt, although few remyelinated axons that could be seen in both groups (Fig. 4G). While we did not observe significant axonal degeneration, nor significant changes in the number of axons within lesions (Fig. 4H), we found that the g-ratio was lower, albeit not significantly, in hiNSC-treated versus PBS-treated *Olig1*^−/−^ mice [0.79 ± 0.010 versus 0.81 ± 0.008 (standard error of the mean)] (Fig. 4I). These data were associated with the finding of fGFP^+^ hiNSCs wrapping around axons within *Olig1*^−/−^ lesions at 6 mpt (Fig. 4J).

These results support the safety, and the potential for long-term integration, of hiNSCs *in vivo*, highlighting their potential therapeutic use in fostering remyelination of chronically demyelinated lesions.

## Discussion

Stem cell transplantation is a promising approach for treating multifactorial neurological disorders.^7^ NSCs have unique benefits, such as immune modulation and trophic support, as well as the potential to replace damaged CNS cells.^19^ However, prior animal models of MS-like diseases demonstrated limited direct remyelination by NSC grafts, as most transplanted cells remained undifferentiated in atypical perivascular niches.^6,8,20^

In this study, we used a focal demyelination model of the spinal cord to study how NSC transplantation affects remyelination after damage. We found that transplanted mouse NSCs and iNSCs stimulated the formation of endogenous mature OLs in wild-type mice as described,^21^ possibly through the secretion of growth factors like BDNF and IGF-1^22^ or by reducing inflammatory myeloid responses.^12^ Importantly, we observed that some transplanted iNSCs also differentiated into astrocytes, which may play a crucial role in promoting CNS-dependent remyelination. This is consistent with evidence that astrocytes support the differentiation of OPCs into mature OLs while inhibiting SC-mediated remyelination.^11^

In addition, we show that iNSC grafts can also directly differentiate into mature OLs within lesions of wild-type mice, potentially driven by SHH, BMPs or WNT intracellular signalling pathways.^23^ However, since this process occurs in competition with the endogenous remyelination response,^24^ we next used *Olig1*^−/−^ mice to abolish graft-host competition in the remyelination process. Using this model of significantly impaired (up to 6 months) endogenous remyelination, we were able to fully establish that transplanted iNSCs can indeed produce mature OLs that effectively remyelinate the CNS.

Using this transgenic model, our study also provides preliminary evidence supporting the remyelination capability of hiNSCs in chronically demyelinated lesions. These results complement previous studies in which other genetic (i.e. shiverer mice) or toxin-induced (i.e. cuprizone) animal models of hypo/demyelination were used to assess the oligodendroglial potential of human stem/precursor cell grafts.^25,26^ Future studies will need to investigate if hiNSCs harbour direct or indirect remyelination ability in wild-type mice and in more physiological models of delayed remyelination (e.g. aged mice).

Although we demonstrate that hiNSCs are safe for transplantation and have the potential of integrating and producing myelin in chronic demyelinated lesions, the use of iNSCs from healthy fibroblast lines limits our ability to study patient-specific traits that may impact their remyelinating efficacy.^27,28^ The limited number of cells retrieved is also a clear limitation, underscoring the need for future research on alternative transplantation doses and immunosuppression protocols. Since our intralesional transplantation approach may not be clinically feasible to treat multiple demyelinating lesions in human neurological disorders, future studies should also investigate alternative delivery methods, such as intrathecal or intracerebroventricular routes.

In conclusion, our findings support the effectiveness of iNSCs as a strategy with minimal immunogenicity and proven immunomodulatory abilities, offering strong evidence for enhanced remyelination driven by the graft, paving the way for future therapeutic applications in regenerative neuroimmunology.

## Supplementary Material

awaf208_Supplementary_Data

## Data Availability

Data supporting the findings of this study are available within the article and its Supplementary material. Raw data are available from the corresponding authors upon request.
