# Correction to: Remyelination of chronic demyelinated lesions with directly induced neural stem cells

**DOI:** 10.1093/brain/awaf274

**Published:** 2025-08-05

Luca Peruzzotti-Jametti, Nunzio Vicario, Giulio Volpe, Sandra Rizzi, Cheek Kwok, Ivan Lombardi, Mads S. Bergholt, Lucas Barea-Moya, Andrea D’Angelo, Alexandra M. Nicaise, Giuseppe D’Amico, Grzegorz Krzak, Cory M. Willis, Sara Gil-Perotin, Olena Hruba, Alice Braga, Molly M. Stevens, José M. Garcia-Verdugo, Kourosh Saeb-Parsy, Chao Zhao, Robin J. M. Franklin, Frank Edenhofer, Stefano Pluchino. Remyelination of chronic demyelinated lesions with directly induced neural stem cells. *Brain*. Published online 7 July 2025. https://doi.org/10.1093/brain/awaf208

The authors apologize for errors in the ‘Funding’ section.

The text has been amended from: ‘Italian Multiple Sclerosis Association (AISM/ FISM) Cod. 2014/PMS/4 to G.V.’ to ‘Italian MS Society Foundation (FISM) Special call Progressive 2014 - Cod. 2014/PMS/2014 to G.V.’; and from ‘AISM/FISM, the Italian Ministry of Health, the ERC, the Medical Research Council, the Bascule Charitable Trust, and the Wellcome Trust-MRC Cambridge Stem Cell Institute to S.P.’ to ‘FISM Special call Progressive 2014 - Cod. 2014/PMS/2014, the Italian Ministry of Health, the ERC, the Medical Research Council, the Bascule Charitable Trust, and the Wellcome Trust-MRC Cambridge Stem Cell Institute to S.P.’.

