# Supplementary material for: Remyelination of chronic demyelinated lesions with directly induced neural stem cells
Source: Brain. 2025 Jul 7;148(10):3505–13. doi: 10.1093/brain/awaf208 (PMC12493045; doi:10.1093/brain/awaf208)
Supplement: awaf208_Supplementary_Data [file awaf208_supplementary_data.pdf]

Supplementary Methods

Cell lines and *in vitro* culture

Mouse NSCs were obtained from the subventricular zone (SVZ) of 7-12 week old (18-20 g) C57BL/6 mice (Charles River, UK) as previously described,[1] under the PPL 80/2457 (to Stefano Pluchino) in line with the Association of Medical Research Charities (AMRC) recommendations.

Briefly, mice were humanely culled by cervical dislocation followed by decapitation, the parietal bones were cut cranially to caudally using micro-surgery scissors, and the brains removed. A brain slice matrix was used to obtain 3 mm thick brain coronal sections starting from 2 mm after the anterior pole of the brain. The SVZ was isolated from coronal sections using iridectomy scissors. Dissected tissues (from at least 2 pooled mice) were transferred to a 15 ml tube with digestion media [early balance salt solution (EBSS, Gibco), papain (1 mg/ml, Worthington), ethylenediaminetetraacetic acid (EDTA) (0.2 mg/ml, Sigma-Aldrich) and L-cysteine (0.2 mg/ml, Sigma-Aldrich)] and incubated for 45 min at 37°C on a rocking platform. At the end of the incubation, the tube was centrifuged at 200g for 12 min, the supernatant was removed, and the pellet was mechanically dissociated with 2 ml of EBSS. The pellet was centrifuged again at 200g for 12 min, further dissociated with a 200 µl pipette, and seeded in complete growth media [CGM: mouse NeuroCult™ basal media (Stem Cell Technologies) plus 1x mouse NeuroCult™ proliferation supplements (Stem Cell Technologies) added with 2 µg/ml heparin (Sigma-Aldrich), 20 ng/ml EGF and 10 ng/ml bFGF]. After approximately 4-7 days, a small percentage of the isolated cells begun to proliferate, giving rise to neurospheres. When neurospheres reached a diameter of 150-200 µm, cells were harvested in a 15 ml tube and centrifuged at 100g for 8 min. The supernatant was removed, and the pellet dissociated by enzymatic digestion with Accumax™ (Ebioscience) at 37°C for 10 min. The number of viable cells was determined by trypan blue exclusion and live cells were re-seeded at clonal density 8,000 cells/cm^2.

Mouse iNSCs were obtained from direct reprogramming of Oct4-GiP Mouse Embryonic Fibroblasts (MEFs) of C57BL/6 mice as previously described.[2] Briefly, Oct4-GiP MEFs were infected with retroviruses encoding for Sox2, Klf4, and c-Myc; iNSCs colonies were then picked at 19 days post infection and cultured as neurospheres in iNSC media [DMEM/F12 (Life Technologies), 1% pen/strep (Invitrogen), 1X N2 (ThermoFisher), 10 ng/ml purified human recombinant EGF (Peprotech), 10 ng/ml human recombinant basic fibroblast growth factor (bFGF, Peprotech)]. New iNSCs media was added to each flask every other day. When

neurospheres reached a diameter of 150-200 µm, cells were collected and harvested in a 15 ml tube (Falcon) and centrifuged at 300g for 8 min. The supernatant was removed, and the cell pellet was dissociated by enzymatic digestion with Accumax™ (Ebioscience) at 37°C for 10 min. The pellet was resuspended in iNSCs media and cells were further centrifuged at 300g for 8 min. The number of viable cells in cell pellets was then determined by trypan blue exclusion and live cells were re-seeded at a density of 9,700 cells/cm^2.

Human iNSCs (hiNSCs) were generated from a commercially available fibroblast cell line (BJ, CRL-2522) using a nonintegrating Sendai virus-based direct conversion strategy, as previously described.[3] Briefly, fibroblasts were seeded at 75,000/well in fibroblast media in a non-coated 12-well plate. On the next day the fibroblasts were transduced using the CytoTune-iPS 2.0 Sendai Reprogramming Kit (Thermo Fisher) with hKOS (MOI: 5), hc-Myc (MOI: 5), hKlf4 (MOI: 3). The day following transfection, the media was switched to neural induction media (NIM) [DMEM:F12 and Neurobasal (1:1), supplemented with N2 supplement (1x, ThermoFisher), 1% glutamax, B27 supplement (1x, ThermoFisher), CHIR99021 (3 µM, Cell Guidance Systems), SB-431542 (2 µM, Cayman Chemicals) and hLIF (10 ng/ml, Cell Signaling Technology)], and cells were moved to 39°C with 5% CO2 to achieve viral clearance over 14 days. A few samples were collected to generate positive controls for quality control assays. Media changes were performed every other day. Following 25 days of transfection, hiNSC colonies were manually selected, seeded onto Growth Factor Reduced (GFR) Matrigel Matrix (1:20 in DMEM/F12) coated plates for expansion, and subjected to quality control assays; hiNSCs were maintained in NIM media until 70% confluent, then lifted using accutase (Sigma Aldrich), spun at 300g for 5 mins, and plated onto GFR-Matrigel coated plates with Y-27632 (10 µM, Miltenyi Biotec) between 1:3-1:5 in NIM media. Media was changed every second day, as needed.

Human iPSC (hiPSCs) were obtained from reprogramming of the same human fibroblast cell line (BJ, CRL-2522) using the StemMACS mRNA Reprogramming Kit (Miltenyi Biotec). Pluripotent colonies were passaged and adapted to feeder-free conditions with hESC matrigel (Corning) and mTeSR1 (STEMCELL Technologies) media, and iPSC media was changed every day. When confluent, cells were lifted using ReLeSR (STEMCELL Technologies) and split onto hESC-matrigel coated plates in mTeSR1 media. For transplantation cells were lifted using accutase (Sigma Aldrich), spun at 300g for 5 mins, and counted.

Lentiviral fGFP tagging

All cells used for transplantation were transduced *in vitro* using a 3rd-generation lentiviral carrier (pRRLsinPPT-hCMV) coding for the enhanced farnesylated-green fluorescent protein (fGFP), which targets the fluorescent protein to the plasma membrane of transduced cells.[4] The functional stability of these cells (in the absence or in the presence of the lentiviral transcript) has been confirmed with clonal and population studies.[5] Briefly, neurospheres from mouse NSCs or mouse iNSCs were harvested, dissociated to a single cells suspension and seeded at high density of $1.5x10^6$ in a T75 cm^2 flask (Sigma-Aldrich) in 5 ml fresh media. After 12 hrs, $3x10^6$ T.U./ml of lentiviral vectors were added, and 6 hrs later additional 5 ml of fresh media were supplemented. 72 hrs after viral transduction, cells were harvested and re-seeded at normal concentration. For human cells, both hiPSCs and hiNSCs were plated at 1 million cells per well in a 6-well plate and given lentivirus at an MOI of 10. After 24 hours media was removed, and cells were allowed to recover for 48 hours. Transgene expression analysis was confirmed by flow cytometry, obtaining >97% $fGFP^+$ cells before transplantation.

Animals, focal demyelination of the spinal cord, and cell transplantation

The wild type (WT) C57BL/6 and NOD SCID mice used in this study were purchased from Jackson laboratory. The Olig1tm1(Cre)Rth (referred to as *Olig1*$^{-/-}$) mice were originally generated by deletion of the majority of the *Olig1* coding region (including the bHLH domain) secondary to the incorporation at the *Olig1* locus of a knock in bacteriophage P1 *Cre* recombinase,[6] and purchased from Jackson laboratory. Animal work was covered by the PPL 70/7715 (to Robin J.M. Franklin) and PPL 70/8702 (to Kourosh Saeb-Parsy) in line with the Association of Medical Research Charities (AMRC) recommendations. All efforts were made to use the fewest number of animals and to minimize suffering of mice utilized.

For mouse stem cell transplantation in the LPC-lesioned spinal cord of WT C57BL/6 mice, we injected a total of 6, 6, and 13 mice with either $1x10^5$ iNSCs, $1x10^5$ NSCs, or PBS, respectively. For mouse stem cell transplantation in the LPC-lesioned spinal cord of Olig1$^{-/-}$ mice, we injected a total of 10 and 12 mice with either $1x10^5$ iNSC or PBS, respectively. For human stem cell transplantation in the LPC-lesioned spinal cord of Olig1$^{-/-}$ mice, we injected a total of 11 and 10 mice with either $1x10^5$ hiNSC or PBS. To avoid hiNSC xenograft rejection, Olig1$^{-/-}$ mice received cyclosporine A (CsA) (SANDIMMUNE) dissolved in the drinking water (at 210 mg/L) from 1 dpl until the end of the experiment.

Ex vivo spinal cord histopathology

For histopathology, mice were deeply anesthetized with an intraperitoneal (i.p.) injection of ketamine 10 mg/ml (Boehringer Ingelheim) and xylazine 1.17 mg/ml (Bayer) in sterile water and transcardially perfused with 1 ml EDTA 5 M in 500 ml saline 0.9% NaCl for 5 min, followed by a solution of 4% PFA in PBS for 5 min. Spinal cords were isolated and post-fixed in 4% PFA in PBS at 4°C overnight. Tissues were then washed in PBS and cryo-protected for at least 48-72 hrs in 30% sucrose in PBS at 4°C. Spinal cords were then embedded in optimum cutting temperature (OCT) media, frozen on liquid nitrogen and cryo-sectioned (20 µm axial section thickness) using a cryostat (CM1850, Leica, Wetzlar, Germany) with a microtome blade (A35, Feather, Osaka, Japan). Sections were then stored at -80°C until use.

For quantification of graft survival, sections were pre-treated with hydrogen peroxide 3% for 15 min and then incubated in blocking solution [10% normal goat serum (NGS, Sigma-Aldrich), 0.1% Triton X100 in PBS] for 1 hr at room temperature. A primary anti-GFP antibody (1:250, Invitrogen) diluted in a solution of PBS, 1% NGS and 0.1% Triton X100 was added to the slides at 4°C overnight. The following day, tissues were washed with PBS and incubated for 1 hr with a biotinylated secondary antibody (1:1,000, Sigma-Aldrich) diluted in a solution of PBS with 1% NGS and 0.1% Triton X100. Components "A" and "B" of Vectastain Elite ABC kit were mixed for 45 min and the reaction developed by means of 3,3'-Diaminobenzidine (DAB) as per manufacturer's instructions. The reaction was blocked dipping the section into distilled water and sections were counterstained with haematoxylin. The tissues were then dehydrated (with increasing alcohol solutions), washed in xylene (Merck, Darnstadt, Germany) and mounted with a synthetic mounting media (EUKITT, Hatfield, PA, USA). The numbers of transplanted $fGFP^+$ cells were calculated on $n \leq 10$ equally spaced axial spinal cord sections using an Olympus BX53 microscope with motorized stage and Neurolucida software (11.07 64-bit, Microbrightfield), which was also used to obtain representative 3D spinal cord reconstructions.

For quantification of graft differentiation, sections were rinsed with PBS and then blocked for 1 hr at room temperature in blocking buffer (10% secondary antibody species serum and 0.1% Triton X100 in PBS). A Fab fragment affinity purified IgG anti-mouse was applied if anti-mouse antibodies were used (1:10, Jackson ImmunoResearch). The following primary antibodies, diluted in blocking buffer, were used at 4°C overnight in a humid chamber: anti-GFP (1:500, Abcam), anti-KI67 (1:250, Abcam), anti-SOX1 (1:50, R&D), anti-GFAP (1:500, Novus Biologicals), anti-NG2 (1:200, Millipore), anti-CC1 (1:200, Calbiochem), anti-TUJ1

(1:500, Biolegend), anti-OLIG2 (1:200, Millipore), anti-OLIG2 (1:200, R&D), anti-periaxin (PRX, 1:1,000; a gift from Professor Peter Brophy, University of Edinburgh), anti-MBP (1:200, Biorad), and anti-NFL (1:500, Abcam). Sections were then washed in PBS with 0.1% Triton X100 and incubated with the appropriate fluorescent secondary antibodies (1:1,000, Alexa Fluor 405, 488, 546, 647, ThermoFisher) for 1 hr at room temperature. After washing in PBS, nuclei were counterstained with DAPI (300 nM, Invitrogen) for 3 min and then mounted with Dako mounting kit (Fluka). Nonspecific staining was controlled in incubations where the primary antibodies were omitted.

Quantification of graft differentiation was obtained from $n \geq 3$ randomized spinal cord regions of interest acquired using a confocal microscope (Leica TCS SP5 Microscope). Data are expressed as % of double or triple positive cells over total $fGFP^+$ cells ± SEM.

Histograms of MBP/NFL intensity were generated using the Fiji ImageJ software (version 2.14.0). After obtaining the single-channel colourful image, the surface plot option was applied with the following settings: “Polygon Multiplier (100%)”, “Shade”, “Fill Plot Background with Black”, and “Smooth”. Then, the representative histogram for a single channel (for example, red) appears and its RGB image was merged with the RGB histogram of another channel of interest (for example, green) previously generated following the same steps.

For quantification of myeloid cells in lesions, sections were processed as above and then stained using a primary antibody anti- IBA1 (1:500, Wako), anti-CD68 (1:200, Invitrogen), and anti-GFP (1:250, Abcam), followed by incubation with the appropriate secondary antibodies, as described above. Quantification of the number of total $IBA1^+$ and $IBA1^+/CD68^+$ cells was obtained from n=2 lesional ROIs of n=2 spinal cord sections per mouse using a confocal microscope (Leica TCS SP5 Microscope). Data are expressed as number of positive cells ± SEM. All quantifications were performed by investigators blinded to the treatment groups.

Polymerase chain reaction (PCR)

Genotypes of $Olig1^{-/-}$ mice were determined by PCR analysis of genomic DNA based on the suggested Jackson Laboratory protocol. Genomic DNA was purified from ear notch samples using DNEasy kit (Qiagen) following manufacturer instructions. A total amount of about 100 ng of DNA was used for subsequent PCR. After PCR, samples were loaded in a 1.2% TBE agarose gel, run was performed at 120 volts for 1 hr and images were recorded using a Bio-Rad ChemiDoc XRS+ system with Image Lab 5.1 software (Bio-Rad).

For PCR analysis of the Oct4 transgene, RNA from iPSCs and iNSCs was isolated using the RNeasy Mini Kit (Qiagen) following manufacturer's instructions. 300 ng of RNA was reverse transcribed using the High Capacity cDNA Reverse Transcription Kit (Thermo Fisher) according to the manufacturer's instructions on a T100 Thermal Cycler (BioRad, 1861096). The RT-PCR reaction was made using DreamTaq buffer (Thermo Fisher), dNTPs (2 mM each, Thermo Fisher), forward and reverse primers (0.5 μM) for Oct4, DreamTaq Hot Start DNA Polymerase (Thermo Fisher), and finished to 24 μL with water. 1 μL of cDNA from each sample was loaded into the PCR reaction. Oct4 (*POU5F1*) primers used: forward – GACAACAATGAGAACCTTCAGGAGA and reverse – CTGGCGCCGGTTACAGAACCA. The reaction was cycled at: 95°C for 3 mins; x30 cycles of 95°C 30 sec, 60°C 30 sec, 72°C 1 min; 72°C 5 min, hold at 4°C. Samples were diluted with Gel Loading Dye (New England BioLabs), and 5 μL was loaded into a 2% agarose gel (Thermo Fisher) in 1X TAE buffer (MP Biomedicals). TrackIt 100 bp DNA Ladder (Thermo Fisher) was used to assess band size. DNA was visualized using Gelred Stain (Biotium) in the gel and imaged on a BioRad XR GelDoc Imager.

Immunoblotting

Western blots were performed on spinal cord tissue samples collected from WT and Olig1$^{-/-}$ mice that were mechanically dissociated and incubated 1 hr at 37°C in lysis buffer and frozen at -80°C until further use. Extracts were defrosted, sonicated and proteins were quantified using Pierce™ BCA Protein Assay kit (Thermo scientific). These samples were then heated at 95°C for 5 min, and an equal amount of proteins were electrophoresed on 12% SDS-PAGE gels and transferred to nitrocellulose membranes. After blocking with 5% non-fat milk in 0.1% PBS-Tween 20 for 1 hr at room temperature, membranes were incubated overnight at 4°C with the following primary antibodies: mouse anti-OLIG1 (1:500, Millipore) and rabbit β-ACTIN (1:10,000, Cell Signaling). After 3 washes in 0.1% PBS-Tween 20, membranes were incubated for 1 hr at room temperature with the appropriate HRP-conjugated secondary antibodies: anti-rabbit HRP conjugated secondary (1:10,000 Thermo Fisher Scientific), anti-mouse HRP conjugated secondary (1:20,000 Thermo Scientific). Protein bands were developed using premixed ready-to-use chemiluminescent HRP detection reagent (Millipore) according to the manufacturer's instructions and acquired using an Uvitec Cambridge Imaging System. The density of each band was quantified using ImageJ analysis software and normalized to housekeeping bands measured in the same membranes.

In situ hybridization

The expression of *Plp* mRNA in demyelinated lesions was examined by *in situ* hybridization with digoxigenin-labelled cRNA probes, using an established protocol.[7] Tissue samples were then analysed using an Olympus BX53 microscope with motorized stage and Neurolucida software (11.07 64-bit, Microbrightfield) to establish the number of *Plp*-positive cells per mm^2 within demyelinating lesions.

Transmission electron microscopy (TEM) and pre-embedding immunogold labelling

For TEM, mice were perfused with 4% glutaraldehyde in PBS containing 0.4 mM CaCl2, and then spinal cords post-fixed at 4°C for 48-72 hrs. The lesion site was sliced into 1 mm-thick slices and incubated overnight at 4ºC with 2% osmium tetroxide (OsO4). The following day, tissues were washed with water and dehydrated using increasing concentration of ethanol (70%, 95%, 100%) for 10 min at room temperature. Slices were then incubated with propylene oxide (BDH - Merck LTD) for 20 min at room temperature. Next, slices were treated with 50% resin (TAAB lab equipment LDT Aldermaston or Durcupan ACM Fluka, Sigma) in propylene oxide for 6 hours at room temperature. After 2 additional overnight incubations at room temperature with 100% resin, tissues were incubated in 100% resin at 60°C until polymerization was completed. Solid resin blocks containing spinal cord samples were semi-thin sectioned (1-1.5 µm) using an Ultracut UC-7 ultramicrotome (Leica, Heidelberg, Germany) and stained with toluidine blue to localize the lesion. Images of toluidine blue-stained sections were acquired using an Olympus BX53 microscope with motorized stage and Neurolucida software (11.07, 64-bit, Microbrightfield). Selected semi-thin sections were then glued to resin blocks and detached from the glass slides through repeated freezing in liquid nitrogen. Ultra-thin sections (70-80 nm) were cut onto copper grids and stained with lead citrate (Reynolds' solution). Imaging was performed using a Hitachi H-600 Transmission Electron Microscope or a FEI Tecnai G2 Spirit Transmission Electron Microscopy (FEI Europe, Eindhoven, Netherlands), equipped with a Morada CCD digital camera (Olympus Soft Imaging Solutions GmbH, Münster, Germany).

For pre-embedding immunogold labelling, spinal cords slices (100 µm-thick) were sectioned using a vibratome and incubated in a primary antibody blocking solution containing 0.3% bovine serum albumin-c (Aurion) and 0.05% sodium azide in 0.1 M phosphate buffer (PB) for

1 hour. Samples were then incubated for 72 hours at 4ºC with the primary antibody (Anti-GFP chicken 1:200 - Aves Lab-) diluted in the blocking solution. After rinsing in 0.1M PB, samples were incubated in a secondary antibody blocking solution containing 0.5% BSAc (Aurion), 0.025% CWFS gelatin (Aurion) and 0.05% sodium azide in 0.1 M PB for 1 hour. This was followed by overnight incubation at 4ºC with the secondary antibody (anti-chicken gold ultrasmall 1:50 -Aurion) diluted in same blocking solution. To enhance gold labelling, silver enhancement (R- GENT SE-LM, Aurion) was performed for 15–25 minutes in the dark, followed by gentle washing in 2% sodium acetate and incubation in gold toning solution (0.05% gold chloride in water) for 10 min. Samples were then washed twice with 0.3% sodium thiosulfate in water. Finally, samples were post-fixed with 2% glutaraldehyde (Electron Microscopy Sciences) in 0.1 M PB for 30 min, rinsed and kept in 0.1M PB containing 0.05% sodium azide at 4ºC until processing them for resin embedding. Immunogold-labelled sections were embedded in resin following the same protocol as for unlabelled samples, except that post-fixation was shortened to 30 minutes and conducted in 1% OsO4 with 7% glucose.

G-ratio quantification was calculated for each axon within the lesion area by dividing axon diameter (inner diameter) by total fiber diameter (outer diameter) and each ratio (sheath thickness) was then coupled with axonal diameter expressed in µm. Linear regression analysis was performed for each group, with all quantified axons considered in the interpolation, and then a mean value was calculated for each group and expressed as violin plot of all quantified axons per group. In addition, for each ROI the total numbers of myelinated and non-myelinated fibers were counted, and the number of axons over lesion area was calculated by dividing the number of viable intact, demyelinated or remyelinated axon in the lesion by the lesion area, and data are expressed as fold change (FC) over WT.

Confocal Raman microspectroscopy

Raman spectroscopy offers label-free insight into the molecular composition of tissues. The confocal Raman microspectroscopy system used to characterize mice spinal cord tissues consists of an upright microscope (Alpha 3000, WITec, GmbH) equipped with a piezoelectric stage. A near-infrared laser (λ_{ex}= 785 nm, Toptica,GmbH) with a maximum output of 250 mW was fiber-coupled into the microscope using a single-mode low OH fiber. All Raman spectra were measured using a Leica 50×/0.70 objective. Using a 100 µm ultralow OH silica fiber acting as a confocal pinhole, the backscattered Raman signals were fed into a high-throughput imaging spectrograph (WITec, GmbH) equipped with a thermoelectrically cooled (−60°C),

charge-coupled device (CCD) camera (IDus, Andor Technology Ltd.). Raman spectra (~3) where measured from the lesion sites. All data were first cropped in the spectral range (800 – 1800 cm^{-1}), with background and autofluorescence subtracted using the "slope function" in the ProjectFour software (WITec, GmbH). Data from PBS-treated WT (n=3), PBS-treated *Olig1*$^{-/-}$ (n=5), and iNSC-treated *Olig1*$^{-/-}$ (n=3) mice were then imported into the Matlab scripting environment (Mathworks, Natick, MA). All the data were normalized to the integrated area to make the spectra comparable across tissues. Myelin index calculated as the lipid (1665 cm^{-1}) to protein (1004 cm^{-1}) ratio from N≥9 ROIs per mouse.

Teratoma Assay

Six days before transplantation, fGFP^{+} hiNSCs were plated at a density of $1x10^5$ per cm^2 and media was exchanged with fresh NIM every other day. Three days before transplantation, fGFP^{+} hiNSCs were given NIM with small molecules (10 ng/ mL hLIF, 3 µM CHIR99021, 2 µM SB431542) for the last time. On the day of surgery, all cells were collected and diluted to 1.6 million cells in 160 µl of 70% Matrigel in NIM or mTeSR1 media, respectively.

NOD SCID mice (56-63 days old) were randomised to receive a local transplantation of fGFP^{+} hiNSCs or fGFP^{+} hiPSCs under the kidney capsule (n= 6 and n= 4, respectively), as previously described.[8] Briefly, an incision of the skin was made near the anatomical position of the kidney. Once the kidney was localized, another incision of the abdominal wall was done to expose it. The kidney was gently pushed out of the abdomen and kept wet with sterile saline. A small incision in the kidney capsule was made with a sharp needle, and cells were injected under the kidney capsule at a concentration of 200,000 cells in 20 µl of 70% Matrigel in NIM (hiNSCs) or 200,000 cells in 20 µl of 70% Matrigel in mTeSR1 (hiPSCs) using a Hamilton syringe. A sterile cotton bud was used to stop bleeding and prevent cell leakage at the point of insertion. The kidney was then placed back, underneath the muscle wall. The muscle wall was sutured with 5–0 vicryl sutures and sutures were used to close the skin layer. At the end, 9 mm autoclip wound clips were placed on the skin to keep the sutures intact.

Mice were deeply anesthetized with an i.p. injection of ketamine 10 mg/ml (Boehringer Ingelheim) and xylazine 1.17 mg/ml (Bayer) in sterile water and transcardially perfused with PBS at 35 (n= 2 fGFP^{+} hiNSCs transplanted mice and n= 1 fGFP^{+} hiPSCs transplanted mouse) or 70 (n= 4 fGFP^{+} hiNSCs transplanted mice and n= 3 fGFP^{+} hiPSCs transplanted mice) days post transplantation (dpt). Kidneys were cryo-protected in 30% sucrose in PBS and embedded in OCT media in liquid nitrogen. OCT embedded kidneys were cryo-sectioned (7 µm coronal

section thickness) onto Superfrost Plus slides using a cryostat (CM1850, Leica, Wetzlar, Germany) with a microtome blade (A35, Feather, Osaka, Japan). Sections were then stored at -80°C until use.

For analysis of kidney structure and identification of the graft, H&E combined with DAB staining was performed. Sections were initially fixed using 4% PFA for 20 minutes then rinsed in PBS. Next, sections were pre-treated with 3% hydrogen peroxide for 15 min and then incubated in blocking solution [10% NGS (Sigma-Aldrich) in 0.1% Triton X100 in PBS] for 1 hr at room temperature. Primary antibody anti-GFP (1:500, Invitrogen) was diluted in a solution of PBS, 1% NGS and 0.1% Triton X100, and incubated at 4°C overnight. The following day, tissues were washed with PBS and incubated for 1 hr with the appropriate secondary biotinylated antibody (1:1,000, Sigma-Aldrich) diluted in a solution of PBS with 1% NGS and 0.1% Triton X100. Components "A" and "B" of the Vectastain Elite ABC kit (Vector Laboratories) were mixed for 45 min and the reaction developed by means of 3,3'-Diaminobenzidine (DAB) as per manufacturer’s instructions. The reaction was blocked dipping the sections into distilled water and subsequently counterstained with haematoxylin and eosin. The tissues were then dehydrated (with increasing alcohol solutions), washed in xylene (Merck, Darnstadt, Germany) and mounted with a synthetic mounting media (EUKITT, Hatfield, PA, USA). The areas of fGFP$^+$ cell masses were calculated on equally distanced kidney sections. fGFP$^+$ cell mass areas were outlined using an Olympus BX53 microscope with motorized stage and Neurolucida software (11.07 64-bit, Microbrightfield). Data are expressed as percent of areas of fGFP$^+$ cells over total area of the kidney.

For the quantification of stem cell graft survival and their differentiation in the kidneys, sections were fixed with 4% PFA solution for 20 min, rinsed with PBS, and then blocked for 1 hr at room temperature in blocking buffer (10% secondary antibody species serum and 0.1% Triton X100 in PBS). The following primary antibodies, diluted in blocking buffer, were used at 4°C overnight in a humid chamber: anti-GFP (1:500, Abcam), anti-FOXA2 (1:500, Abcam), anti-αSMA (1:500, Abcam) and anti-NESTIN (1:100, Abcam). Sections were then washed in PBS with 0.1% Triton X100 and incubated with the appropriate fluorescent secondary antibodies (1:1,000, Alexa-fluor 488, 546, 647, Invitrogen) for 1 hr at room temperature. After washing in PBS, nuclei were counterstained with DAPI (300 nM, Invitrogen) for 5 min and then mounted with EverBrite mounting media (Biotium).

Statistical analyses

Statistical differences among groups were analysed with GraphPad Prism (version 5.00 for Mac, GraphPad Software) using the appropriate statistical tests (Two-way ANOVA with Tukey's multiple comparisons test when comparing >2 groups and >1 condition; One-way ANOVA with Holm-Šídák's multiple comparison test when comparing >2 groups and 1 condition; Unpaired t-test when comparing 2 groups and 1 condition). Teratoma formation was analysed via a Kaplan Meier curve and Log-rank test. All values are expressed as mean ± SEM and a p-value ≤ 0.05 was consider as significant in all analyses.

Declaration of Generative AI in the writing process

The authors used Google Gemini to check the grammar. After using this tool/service, the authors reviewed the content and take full responsibility for the content of the publication.

References

1. Vescovi AL, Snyder EY. Establishment and properties of neural stem cell clones: plasticity in vitro and in vivo. *Brain Pathol*. Jul 1999;9(3):569-98.
2. Thier M, Worsdorfer P, Lakes YB*, et al*. Direct Conversion of Fibroblasts into Stably Expandable Neural Stem Cells. *Cell stem cell*. Apr 6 2012;10(4):473-479. doi:Doi 10.1016/J.Stem.2012.03.003
3. Meyer S, Worsdorfer P, Gunther K, Thier M, Edenhofer F. Derivation of Adult Human Fibroblasts and their Direct Conversion into Expandable Neural Progenitor Cells. *J Vis Exp*. Jul 29 2015;(101):e52831. doi:10.3791/52831
4. Follenzi A, Ailles LE, Bakovic S, Geuna M, Naldini L. Gene transfer by lentiviral vectors is limited by nuclear translocation and rescued by HIV-1 pol sequences. *Nat Genet*. Jun 2000;25(2):217-22. doi:10.1038/76095
5. Pluchino S, Quattrini A, Brambilla E*, et al*. Injection of adult neurospheres induces recovery in a chronic model of multiple sclerosis. *Nature*. Apr 17 2003;422(6933):688-694. doi:10.1038/nature01552
6. Lu QR, Sun T, Zhu ZM*, et al*. Common developmental requirement for Olig function indicates a motor neuron/oligodendrocyte connection. *Cell*. Apr 5 2002;109(1):75-86. doi:Doi 10.1016/S0092-8674(02)00678-5
7. Kotter MR, Li WW, Zhao C, Franklin RJ. Myelin impairs CNS remyelination by inhibiting oligodendrocyte precursor cell differentiation. *J Neurosci*. Jan 4 2006;26(1):328-32. doi:10.1523/JNEUROSCI.2615-05.2006
8. Georgakopoulos N, Prior N, Angres B*, et al*. Long-term expansion, genomic stability and in vivo safety of adult human pancreas organoids. *BMC developmental biology*. Feb 26 2020;20(1):4. doi:10.1186/s12861-020-0209-5

Supplemental Figure Legends

Figure S1. Mouse iNSCs and NSCs show a similar differentiation profile after transplantation in LPC spinal cord lesions of WT C57BL/6 mice.

(A-B) Representative images (A) and quantification (B) of the immunoreactivity of transplanted mouse fGFP$^+$ iNSCs and NSCs for the stem cell marker SOX1, the proliferation marker KI67, the astroglial lineage marker GFAP, and the neuronal marker TUJ1 at 21 dpl. Data are mean percentage ± SEM over total fGFP$^+$ analysed in n= 3 mice/group. P-value: **≤ 0.01, ****≤ 0.0001; $$$$≤ 0.0001 *vs* SOX1$^+$ iNSCs; ####≤ 0.0001 *vs* SOX1$^+$ NSCs. Two-way ANOVA, Tukey's multiple comparisons test.

(C-D) Representative images (C) and quantification (D) of the immunoreactivity of transplanted mouse fGFP$^+$ iNSCs and NSCs for the oligodendroglial progenitor markers OLIG2/NG2, and the mature oligodendrocytes markers OLIG2/CC1 at 10 and 21 dpl. P-value: **≤ 0.01, ***≤ 0.001. Two-way ANOVA, Tukey's multiple comparisons test.

Dpl: days post lesion.

Figure S2. *Olig1*$^{-/-}$ mice and mouse iNSCs transplantation.

(A) Genomic PCR analysis for the *Olig1* and *Cre* genes in and *Olig1*$^{-/-}$ mice and WT C57BL/6 controls.

(B) Representative blot of OLIG1 protein expression levels in the spinal cord of WT and Olig1$^{-/-}$ mice with quantification over β-actin expression levels. N.D.: Not Detected. Data are mean ± SEM from n= 4 mice/group.

(C) Quantification and representative images of transplanted mouse fGFP$^+$ iNSCs immunoreactive for the stem cell marker SOX1, the proliferation marker KI67, the astroglial lineage marker GFAP, the neuronal marker TUJ1, the oligodendroglial progenitor markers OLIG2/NG2, and the mature oligodendrocytes markers OLIG2/CC1 at 21 dpl. Data are mean percentage ± SEM over total fGFP$^+$ cells analysed in n= 3 mice. P-value: *≤0.05.

P-value: *≤ 0.05, **≤ 0.01, ***≤ 0.001; $$$$≤ 0.0001 *vs* SOX1$^+$ iNSCs. One-way ANOVA, Holm-Šídák's multiple comparison test.

Dpl: days post lesion.

Figure S3. Myelin index of LPC spinal cord lesions.

Raman microspectroscopy-based myelin index calculated as the lipid (1665 cm^{-1}) to protein (1004 cm^{-1}) ratio. P-value: *≤ 0.05. One way ANOVA, Holm-Šídák's multiple comparisons

test. N≥9 ROIs per mouse from PBS-treated WT (n=3), PBS-treated *Olig1*$^{-/-}$ (n=5), and iNSC-treated *Olig1*$^{-/-}$ (n=3) mice.

Figure S4. *In vitro* derivation and characterisation of hiNSCs before transplantation.

(A) Schematic representation of hiNSCs generation, expansion, and preparation for transplantation. NIM: neural induction media.

(B) Flow cytometry analysis of hiNSCs transduced with a third-generation lentiviral vector coding for the fGFP prior transplantation.

(C) Genomic PCR analysis for the *Oct4* transgene in hiNSCs and hiPSCs controls.

(D) Representative images of fGFP$^+$ hiNSCs cultured *in vitro* in NIM and stained for GFAP, SOX1, SOX2, SSEA4, Olig2, NeuN, Oct4, and TUJ1. Nuclei are counterstained with DAPI.

Figure S5. hiNSCs are not teratogenic in NOD SCID mice.

(A) Representative images of kidneys extracted from NOD SCID mice showing teratoma formation in hiPSC-treated mice only.

(B) Representative images of fGFP$^+$ hiPSCs cells at 70 dpt in the kidney capsule of NOD SCID mice immunostained for markers of the three germinal layers (FOXA2: endoderm, Nestin: ectoderm, and αSMA: mesoderm).

(C) DAB and Haematoxylin-stained representative image of a hiPSC injected kidney (left) and quantification of fGFP$^+$ cell area (right) in the kidney capsule of NOD SCID mice transplanted with either hiPSCs (n=3) or hiNSCs (n=4) at 70 dpt. Data are percentage of fGFP$^+$ area over total area of the kidney. P-value *≤0.05. Unpaired t-test.

Dpt: days post transplantation.

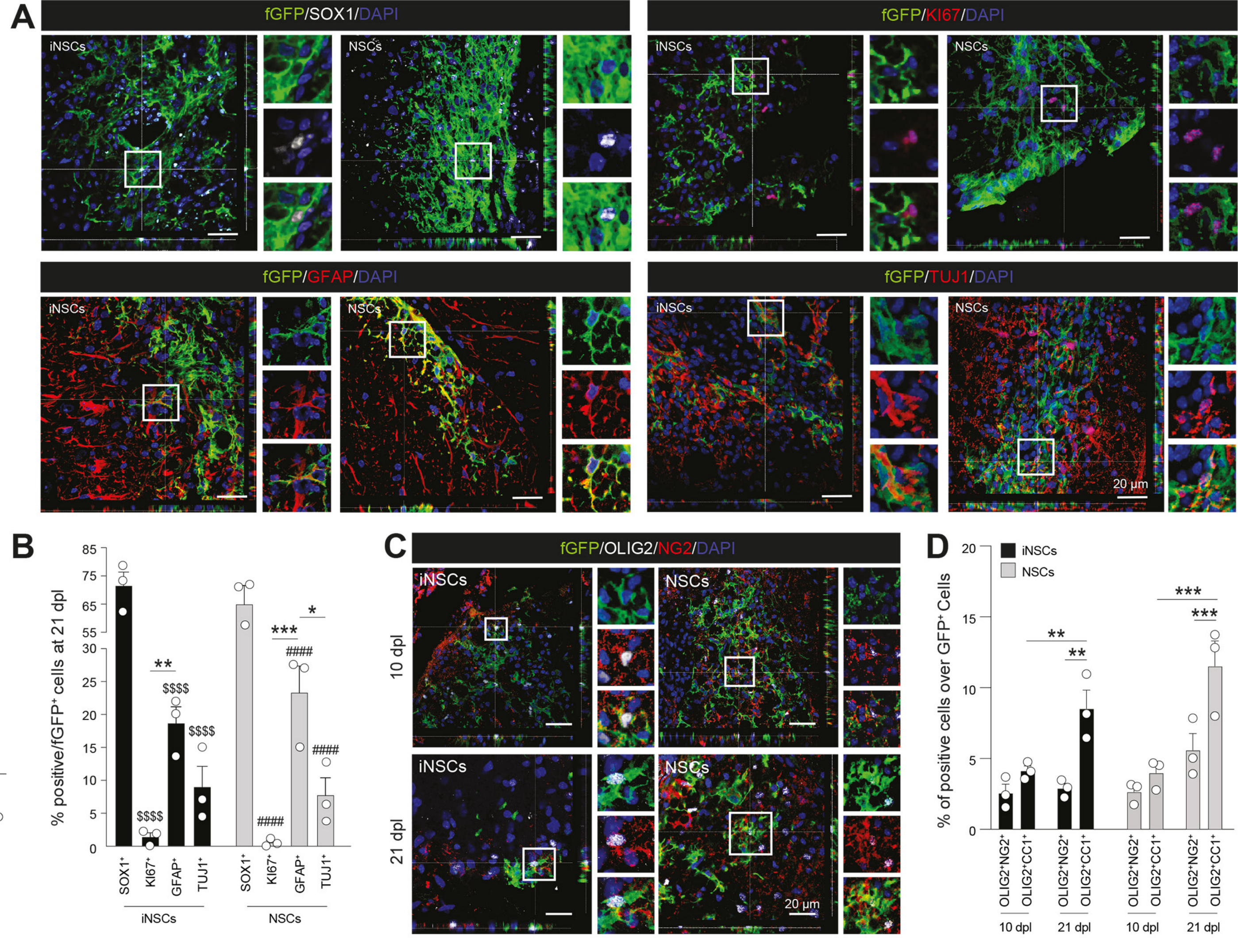

A
fGFP/SOX1/DAPI
iNSCs
NSCs
fGFP/KI67/DAPI
iNSCs
NSCs
fGFP/GFAP/DAPI
iNSCs
NSCs
fGFP/TUJ1/DAPI
iNSCs
NSCs
20 µm
B
% positive/fGFP⁺ cells at 21 dpl
85
75
65
55
30
25
20
15
10
5
0
**
$$$$
$$$$
$$$$
$$$$
***
*
####
####
####
SOX1⁺
KI67⁺
GFAP⁺
TUJ1⁺
SOX1⁺
KI67⁺
GFAP⁺
TUJ1⁺
iNSCs
NSCs
C
fGFP/OLIG2/NG2/DAPI
iNSCs
NSCs
10 dpl
21 dpl
iNSCs
NSCs
20 µm
D
iNSCs
NSCs
% of positive cells over GFP⁺ Cells
20
15
10
5
0
**
**
***
***
OLIG2⁺NG2⁺
OLIG2⁺CC1⁺
OLIG2⁺NG2⁺
OLIG2⁺CC1⁺
OLIG2⁺NG2⁺
OLIG2⁺CC1⁺
OLIG2⁺NG2⁺
OLIG2⁺CC1⁺
10 dpl
21 dpl
10 dpl
21 dpl

Figure S1

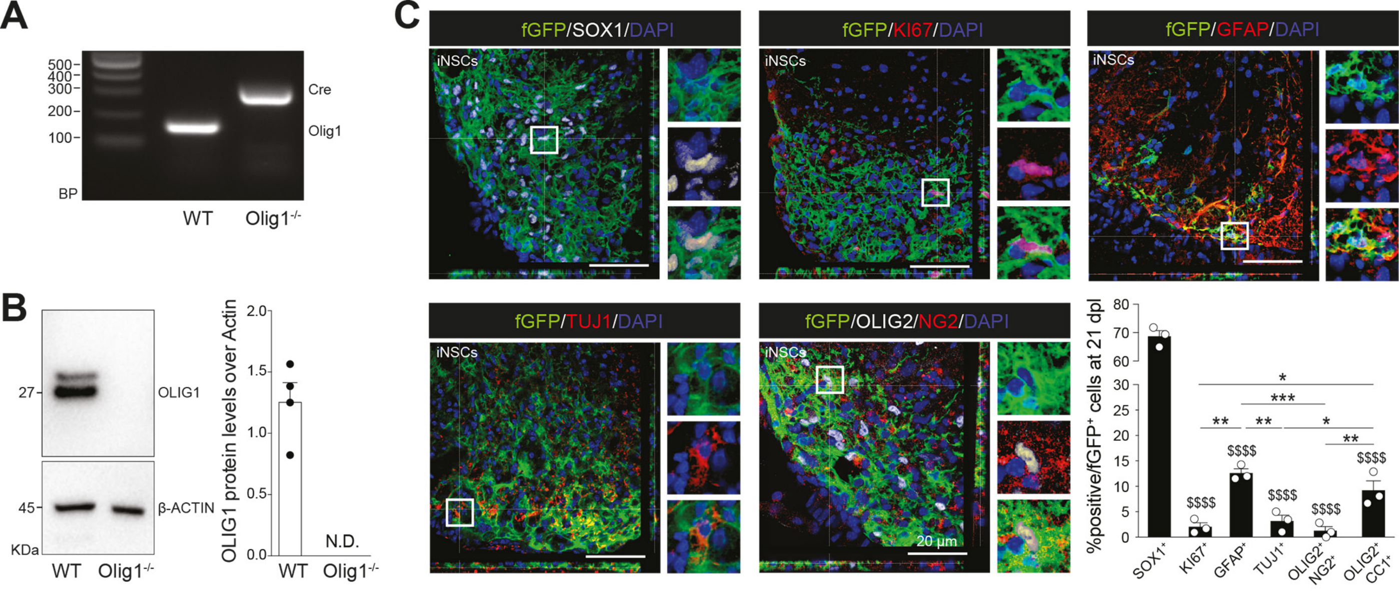
A
500
400
300
200
100
BP
Cre
Olig1
WT
Olig1-/-
B
27
45
KDa
OLIG1
β-ACTIN
WT
Olig1-/-
OLIG1 protein levels over Actin
2.0
1.5
1.0
0.5
0.0
N.D.
WT
Olig1-/-
C
fGFP/SOX1/DAPI
fGFP/KI67/DAPI
fGFP/GFAP/DAPI
fGFP/TUJ1/DAPI
fGFP/OLIG2/NG2/DAPI
iNSCs
20 µm
%positive/fGFP+ cells at 21 dpl
SOX1+
KI67+
GFAP+
TUJ1+
OLIG2+ NG2+
OLIG2+ CC1+
$$$$
*
**
***

Figure S2

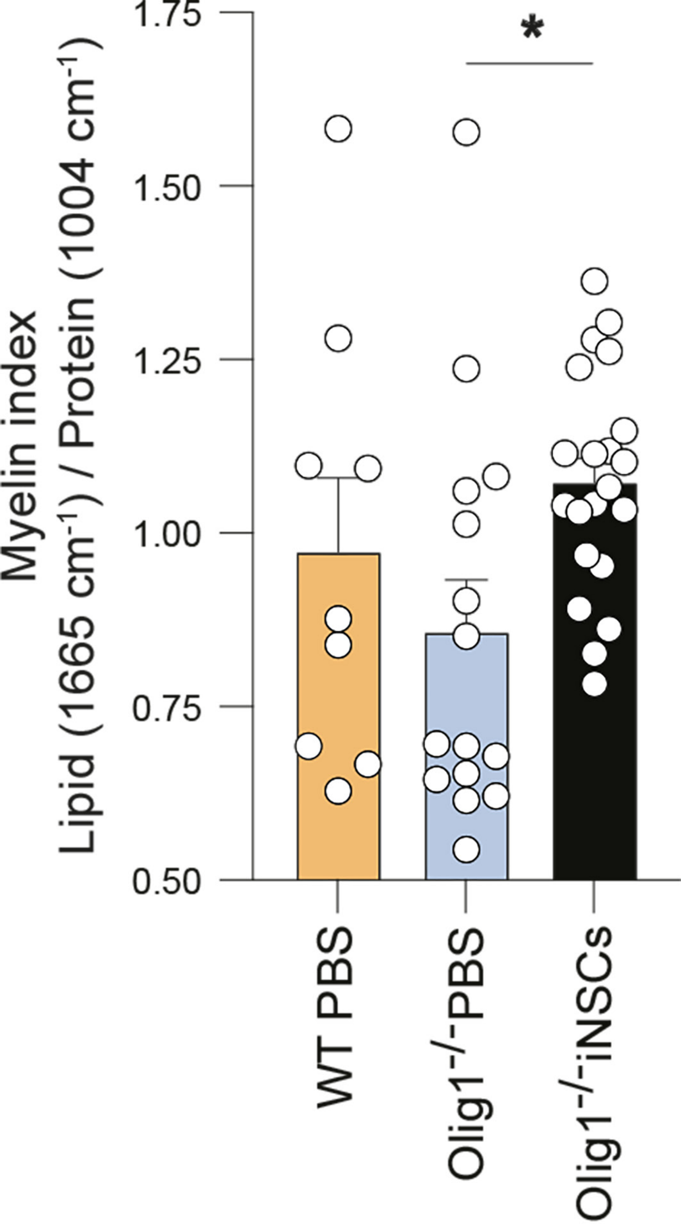

Myelin index
Lipid (1665 cm-1) / Protein (1004 cm-1)
1.75
1.50
1.25
1.00
0.75
0.50
*
WT PBS
Olig1-/-PBS
Olig1-/-iNSCs

Figure S3

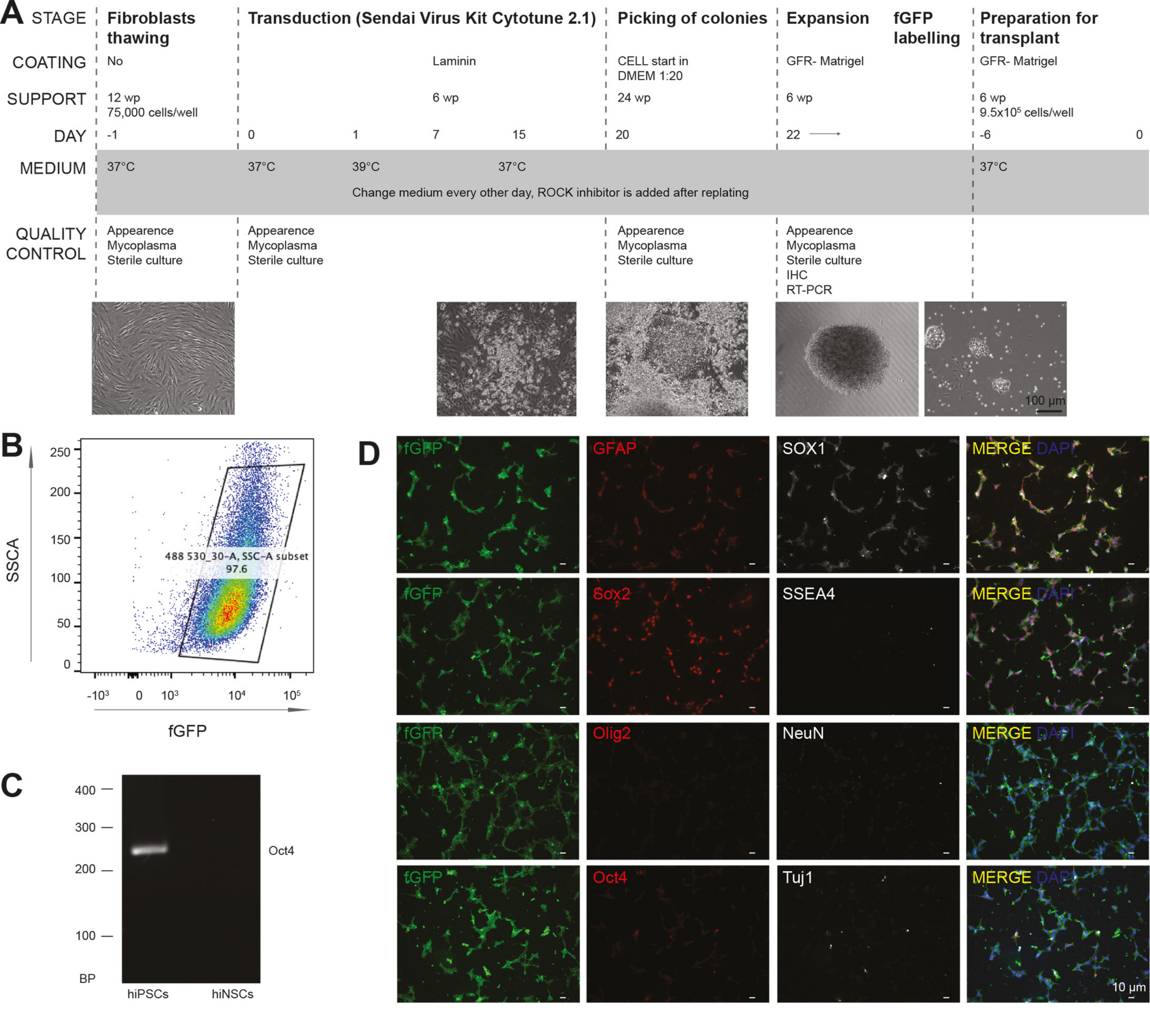
A
STAGE
Fibroblasts thawing
Transduction (Sendai Virus Kit Cytotune 2.1)
Picking of colonies
Expansion
fGFP labelling
Preparation for transplant
COATING
No
Laminin
CELL start in DMEM 1:20
GFR- Matrigel
GFR- Matrigel
SUPPORT
12 wp
75,000 cells/well
6 wp
24 wp
6 wp
6 wp
9.5x10^5 cells/well
DAY
-1
0
1
7
15
20
22
-6
0
MEDIUM
37°C
37°C
39°C
37°C
37°C
Change medium every other day, ROCK inhibitor is added after replating
QUALITY CONTROL
Appearence
Mycoplasma
Sterile culture
Appearence
Mycoplasma
Sterile culture
Appearence
Mycoplasma
Sterile culture
Appearence
Mycoplasma
Sterile culture
IHC
RT-PCR
100 µm
B
SSCA
250
200
150
100
50
0
488 530_30-A, SSC-A subset
97.6
-10^3
0
10^3
10^4
10^5
fGFP
C
400
300
200
100
BP
Oct4
hiPSCs
hiNSCs
D
fGFP
GFAP
SOX1
MERGE DAPI
fGFP
Sox2
SSEA4
MERGE DAPI
fGFP
Olig2
NeuN
MERGE DAPI
fGFP
Oct4
Tuj1
MERGE DAPI
10 µm

Figure S4

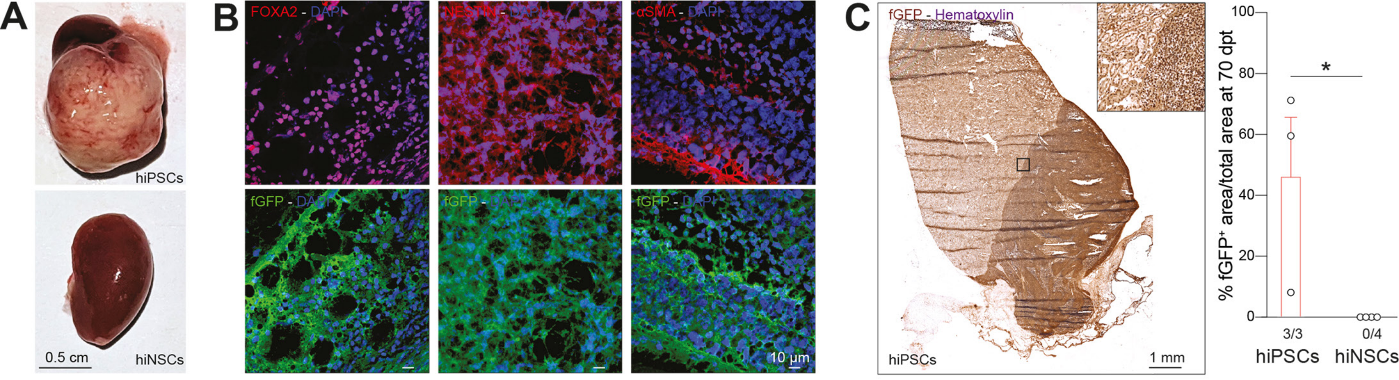

A
hiPSCs
0.5 cm
hiNSCs
B
FOXA2 - DAPI
NESTIN - DAPI
αSMA - DAPI
fGFP - DAPI
fGFP - DAPI
fGFP - DAPI
10 µm
C
fGFP - Hematoxylin
hiPSCs
1 mm
% fGFP+ area/total area at 70 dpt
100
80
60
40
20
0
*
3/3
0/4
hiPSCs
hiNSCs

Figure S5

Cell type/Nomenclature	Origin	N of clones	N of transplanted mice per treatment group
NSCs	Mouse subventricular zone (isolated from C57BL/6 mice)	1	WT C57BL/6 mice sacrificed at 10 dpl = 3
			WT C57BL/6 mice sacrificed at 21 dpl = 3
iNSCs	Direct reprogramming of Mouse Embryonic Fibroblasts (isolated from C57BL/6 mice)	1	WT C57BL/6 mice sacrificed at 10 dpl = 3
			WT C57BL/6 mice sacrificed at 21 dpl = 3
			Olig1-/- mice sacrificed at 21 dpl = 3-4 (IF-insitu); 3 (TEM); 3 (Raman)
hiNSCs	Direct reprogramming of commercially available human fibroblast cell line BJ, CRL-2522	1	Olig1-/- mice sacrificed at 1.5 mpt = 4
			Olig1-/- mice sacrificed at 6 mpt = 4 (IF); 3 (TEM)
			NOD SCID mice = 6
hiPSCs	Reprogramming of commercially available human fibroblast cell line BJ, CRL-2522	1	NOD SCID mice = 4

Supplementary Table 1

dpl= days post lesion

mpt= months post lesion
